# Supplementary figures and images for: The Leaf Color and Trichome Density Influence the Whitefly Infestation in Different Cassava Cultivars
Source: Insects. 2022 Dec 21;14(1):4. doi: 10.3390/insects14010004 (PMC9865453; doi:10.3390/insects14010004)

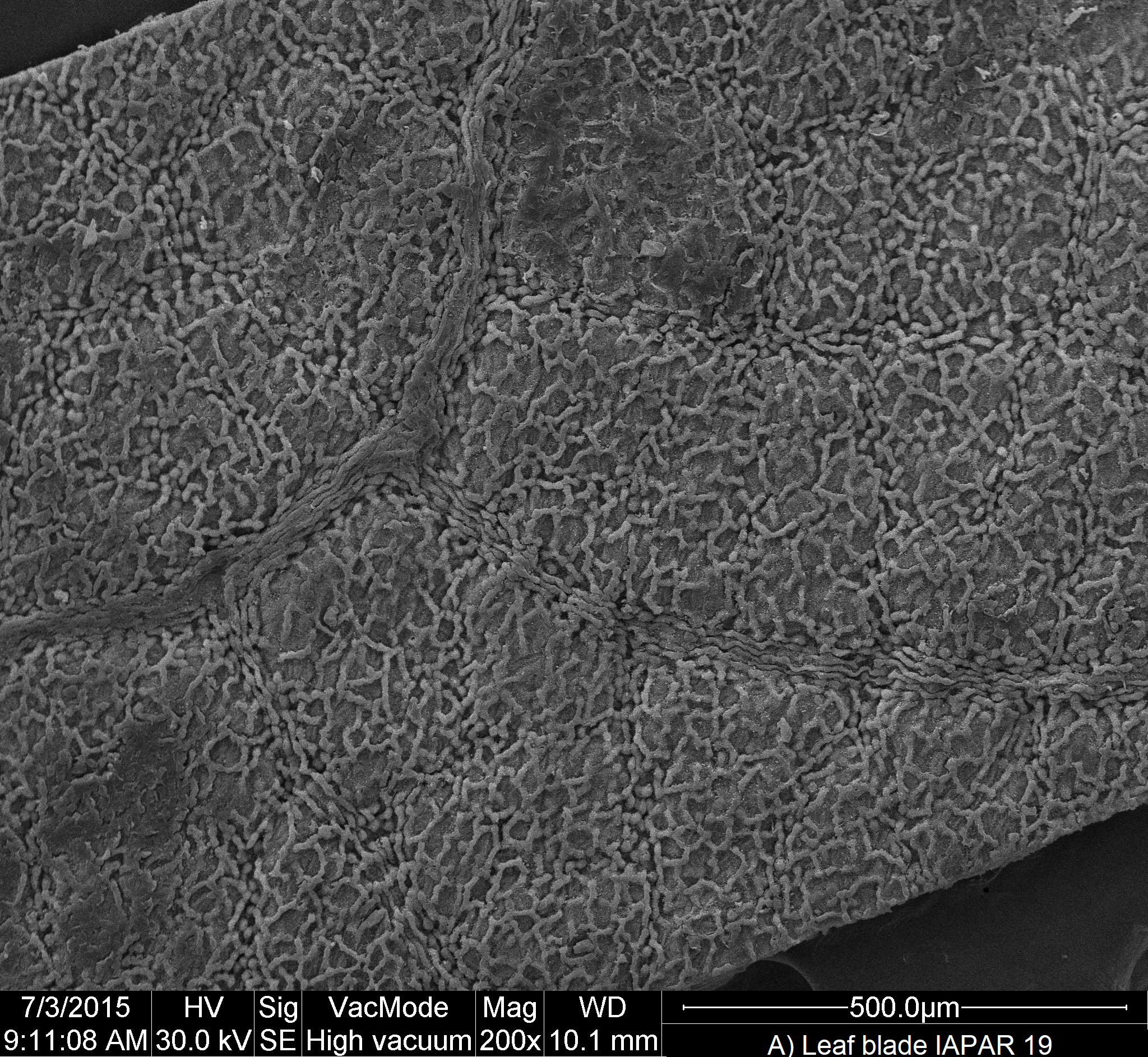

Supplement: Supplementary file 1 [file insects-14-00004-s001.zip › File S1/A) Leaf blade IAPAR 19 - Shoot leaf.tif]

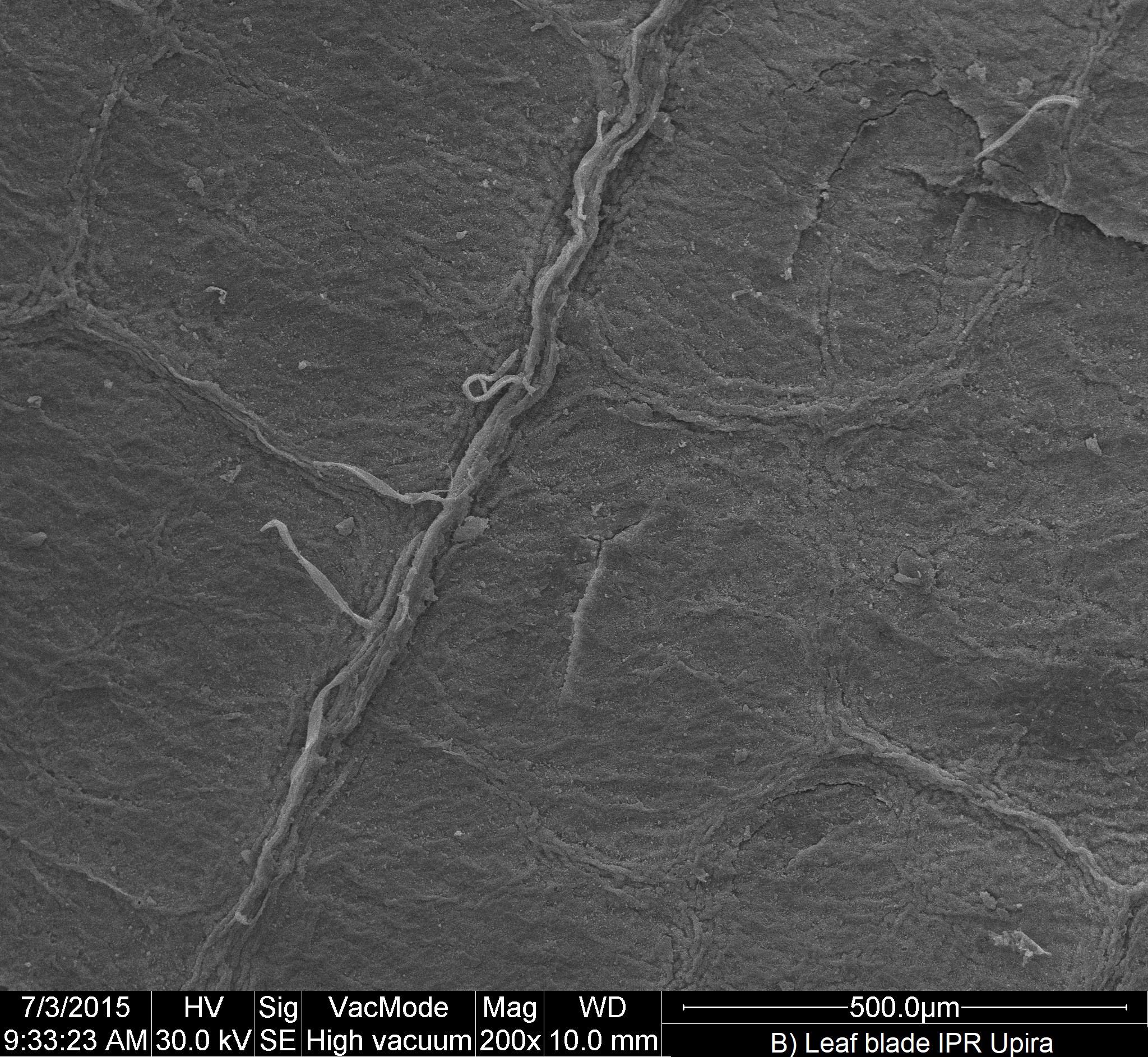

Supplement: Supplementary file 1 [file insects-14-00004-s001.zip › File S1/B) Leaf blade IPR Upira - Shoot leaf.tif]

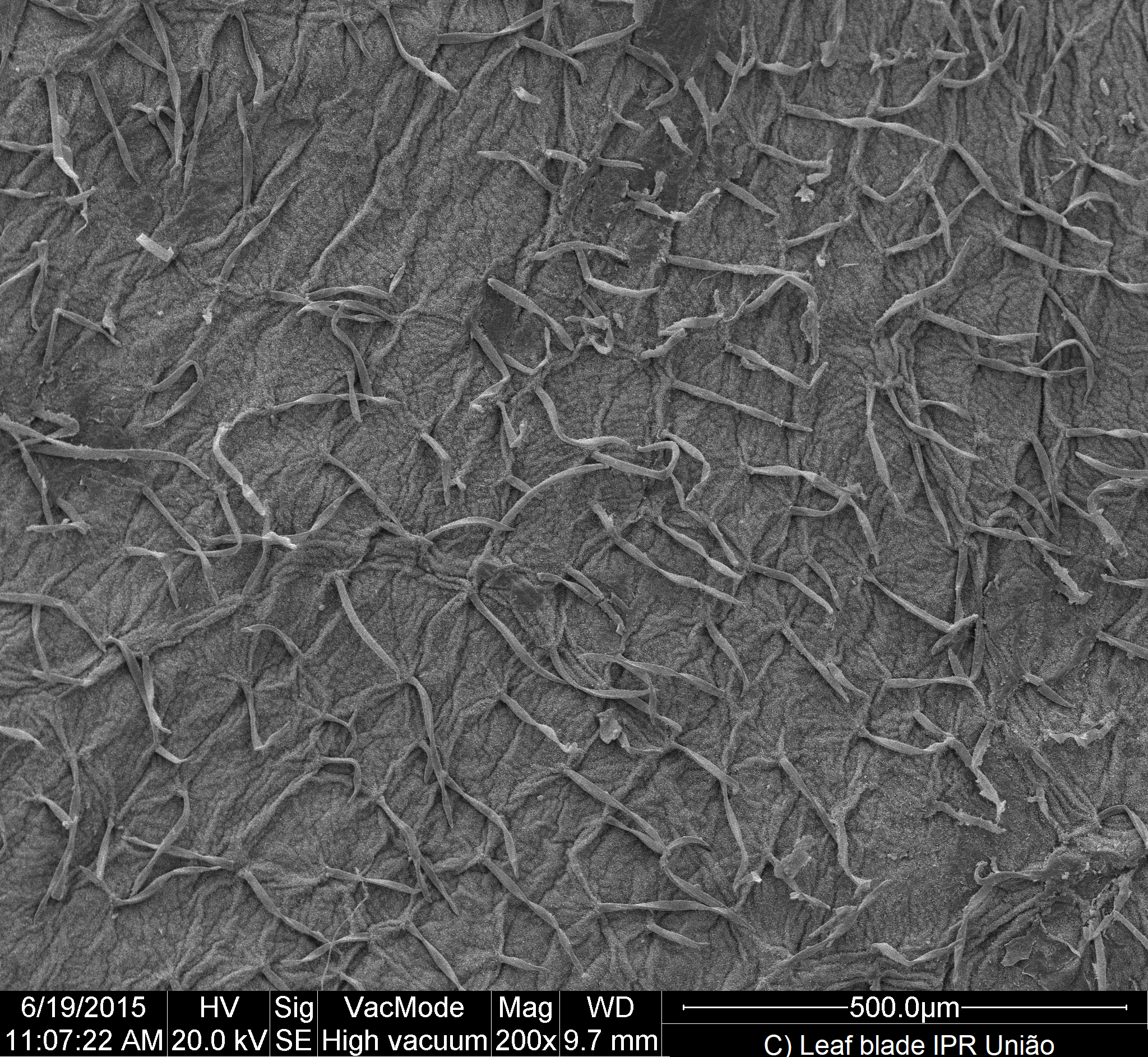

Supplement: Supplementary file 1 [file insects-14-00004-s001.zip › File S1/C) Leaf blade IPR Uni╞o - Shoot leaf.tif]

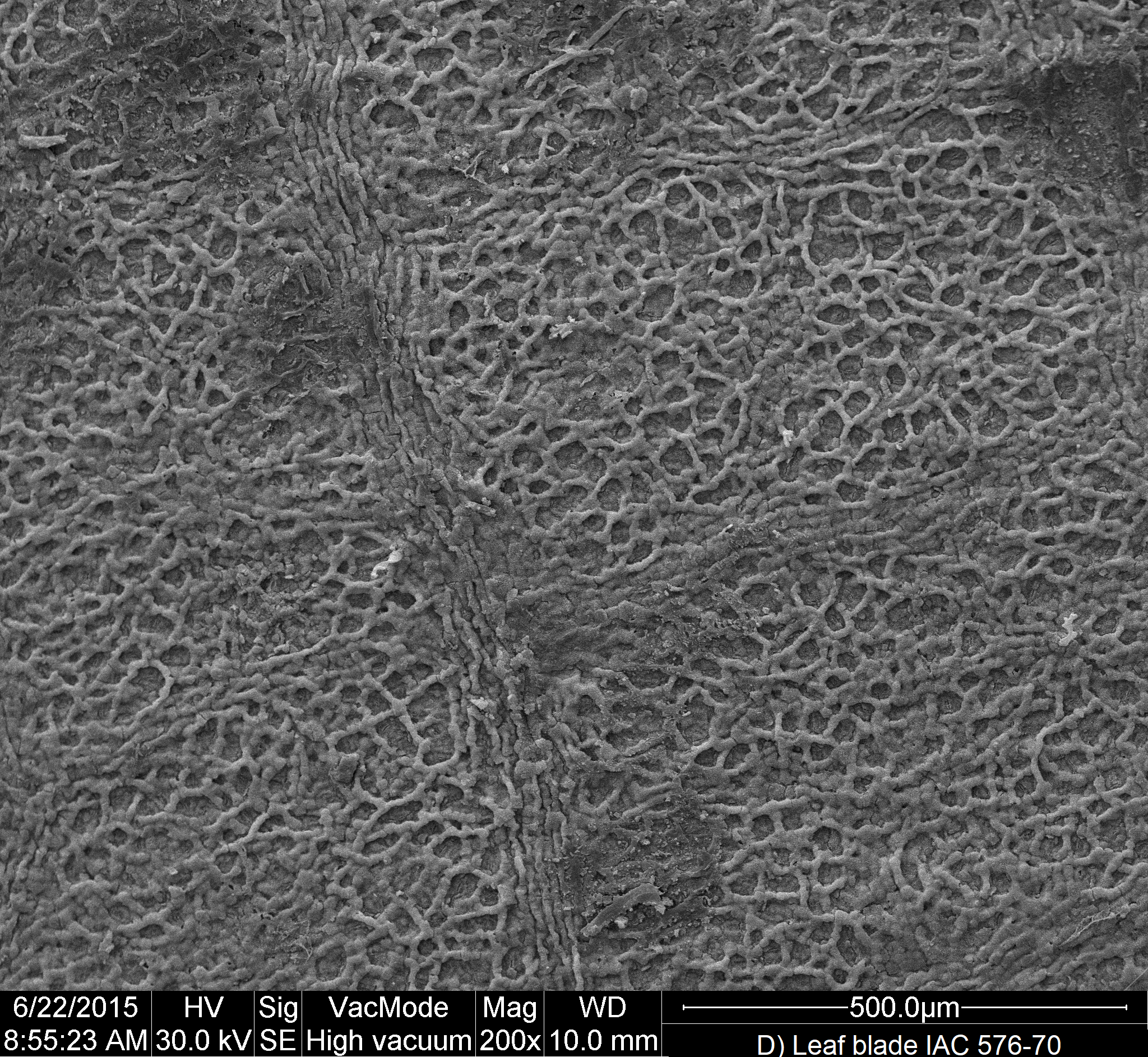

Supplement: Supplementary file 1 [file insects-14-00004-s001.zip › File S1/D) Leaf blade IAC 576-70 - Shoot leaf.tif]

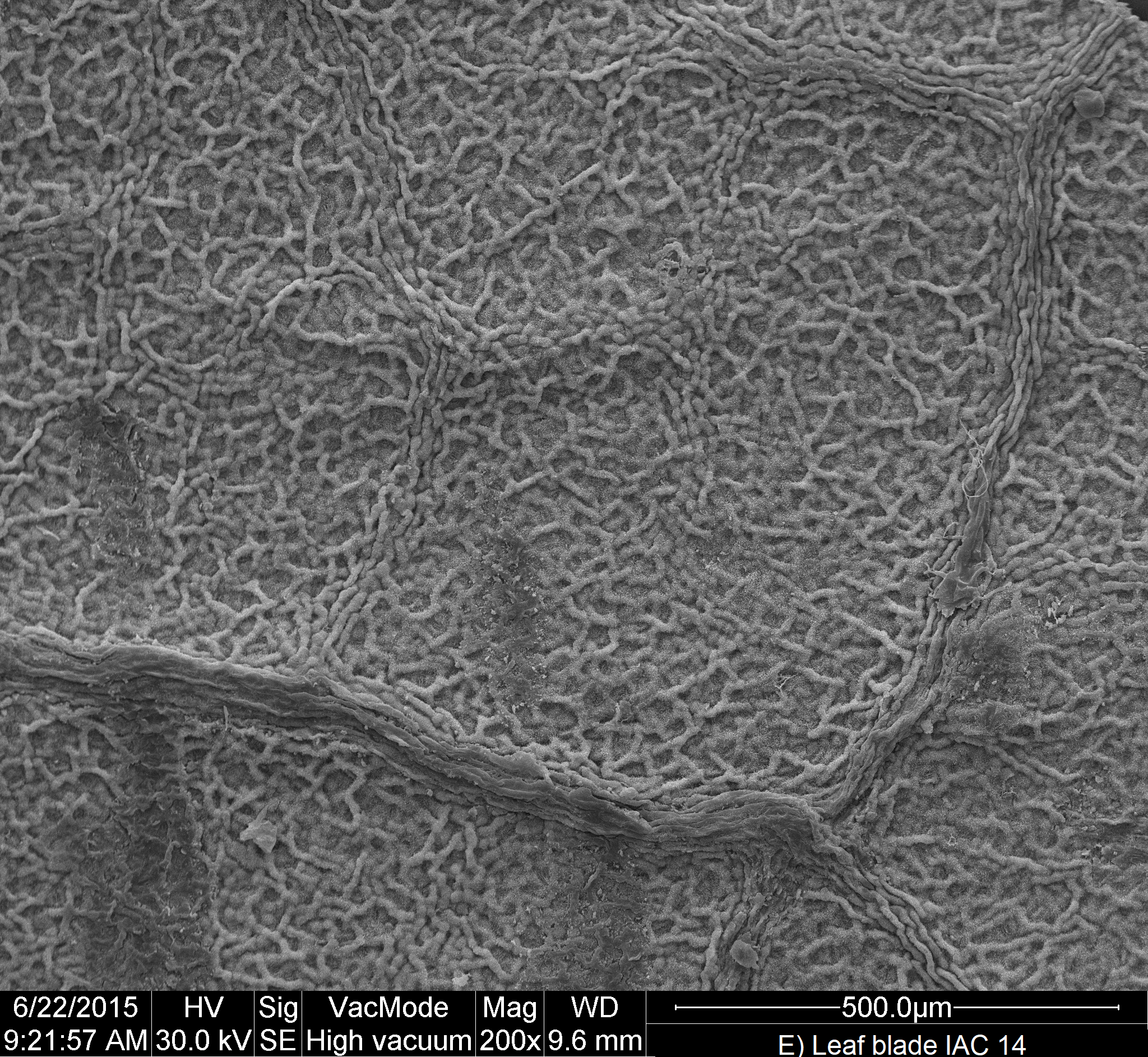

Supplement: Supplementary file 1 [file insects-14-00004-s001.zip › File S1/E) Leaf blade IAC 14 - Shoot leaf.tif]

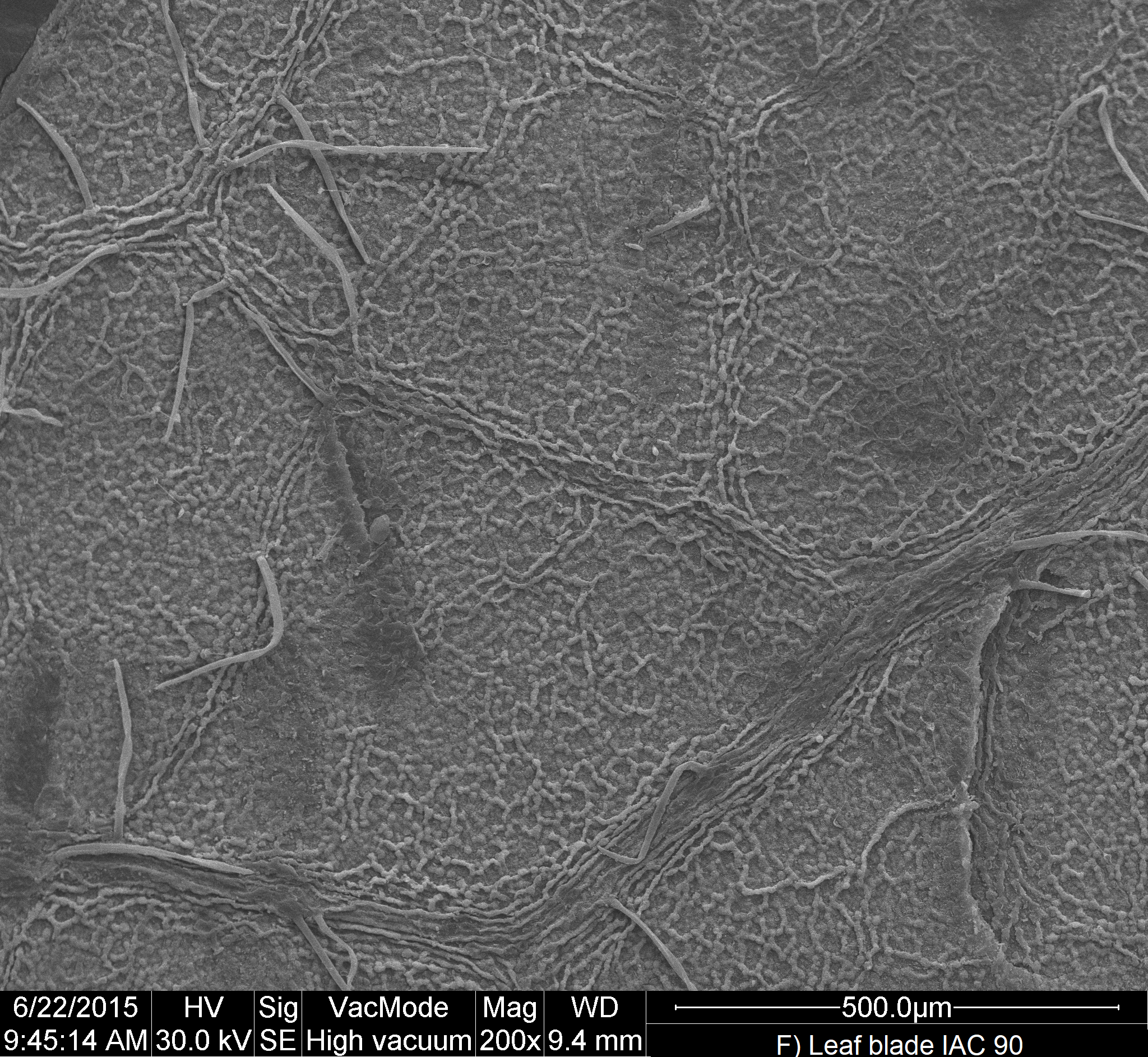

Supplement: Supplementary file 1 [file insects-14-00004-s001.zip › File S1/F) Leaf blade IAC 90 - Shoot leaf.tif]

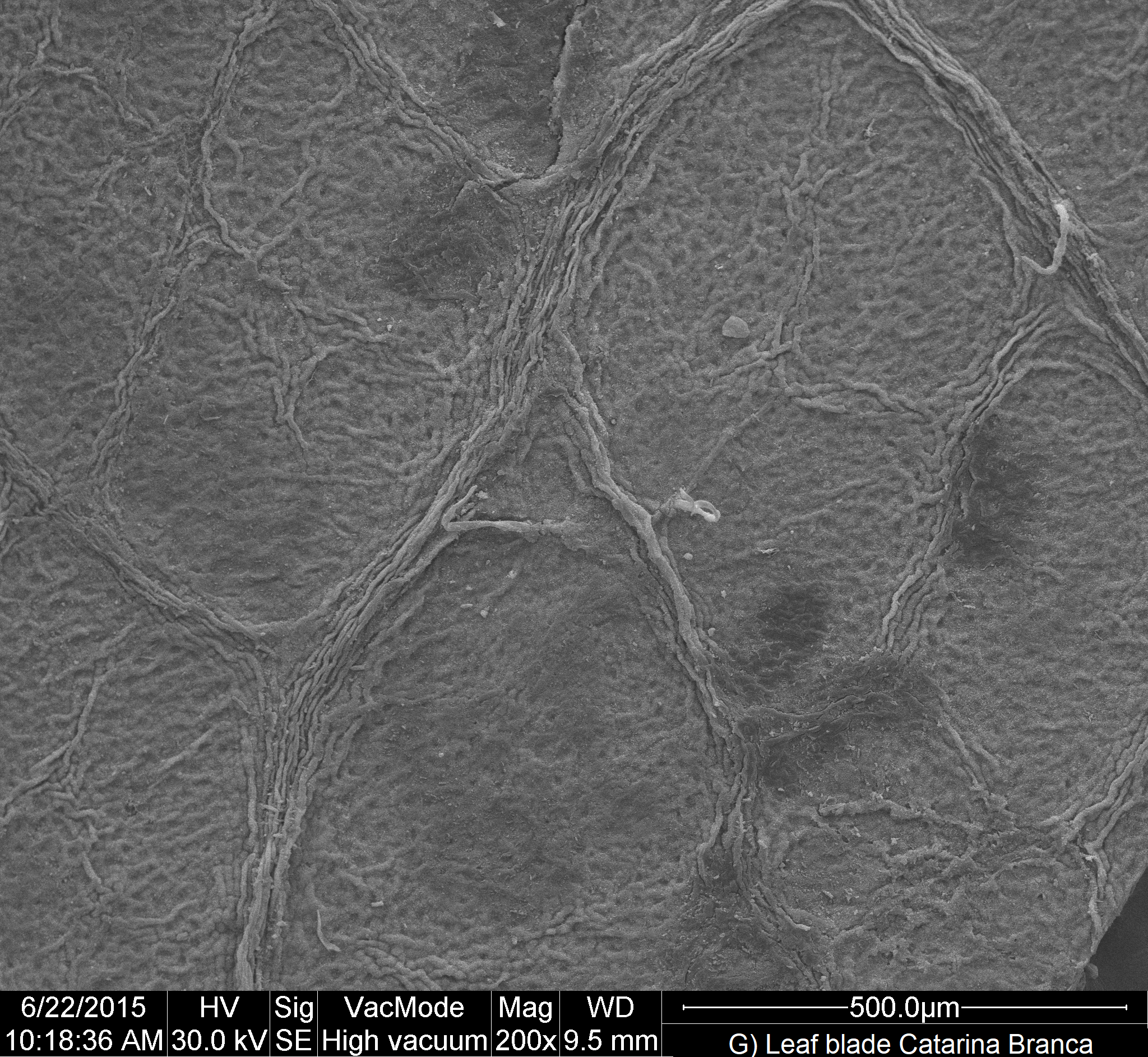

Supplement: Supplementary file 1 [file insects-14-00004-s001.zip › File S1/G) Leaf blade Catarina Branca - Shoot leaf.tif]

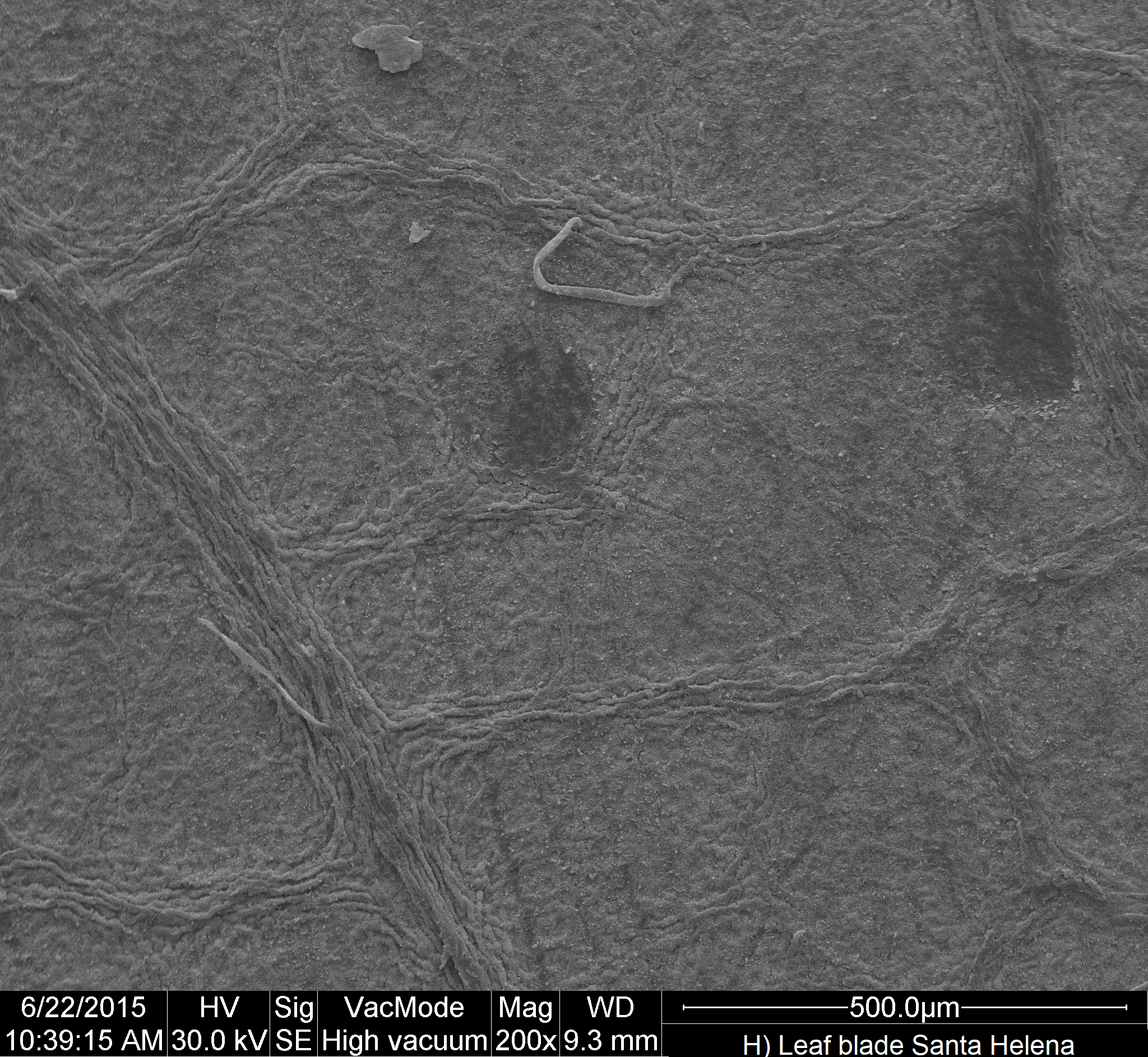

Supplement: Supplementary file 1 [file insects-14-00004-s001.zip › File S1/H) Leaf blade Santa Helena - Shoot leaf.tif]

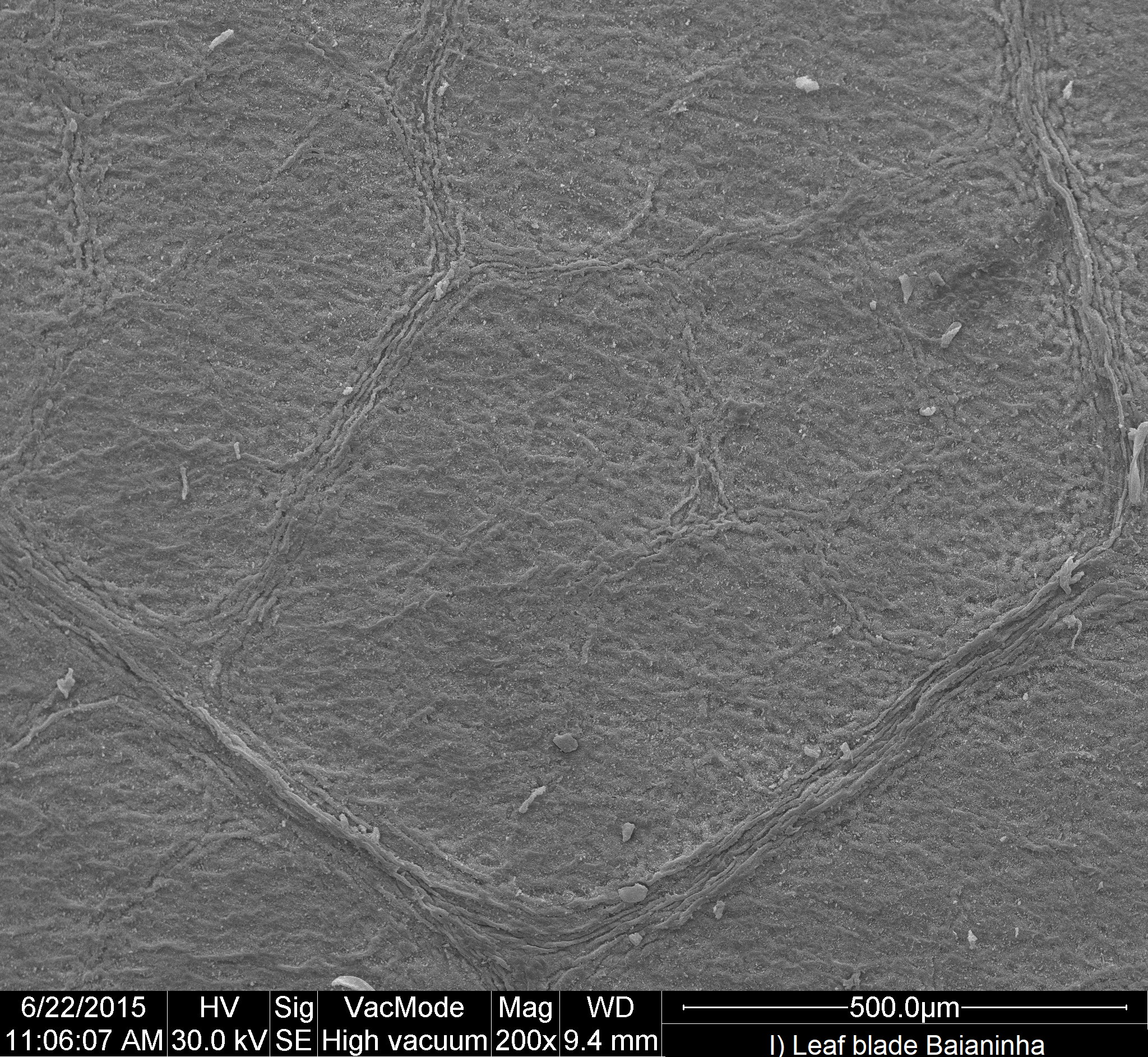

Supplement: Supplementary file 1 [file insects-14-00004-s001.zip › File S1/I) Leaf blade Baianinha - Shoot leaf.tif]

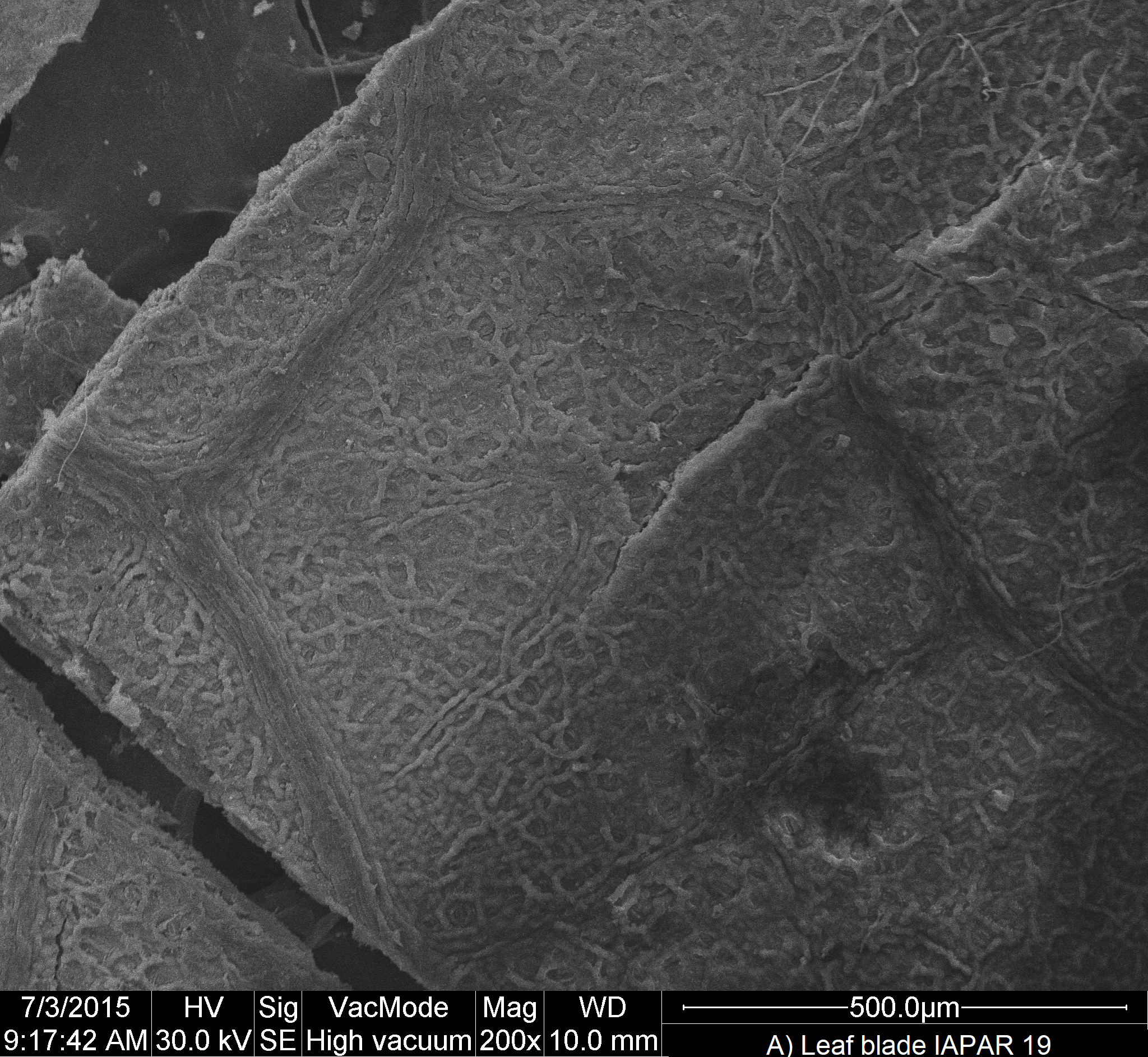

Supplement: Supplementary file 1 [file insects-14-00004-s001.zip › File S2/A) Leaf blade IAPAR 19 - Superior third portion leaf.tif]

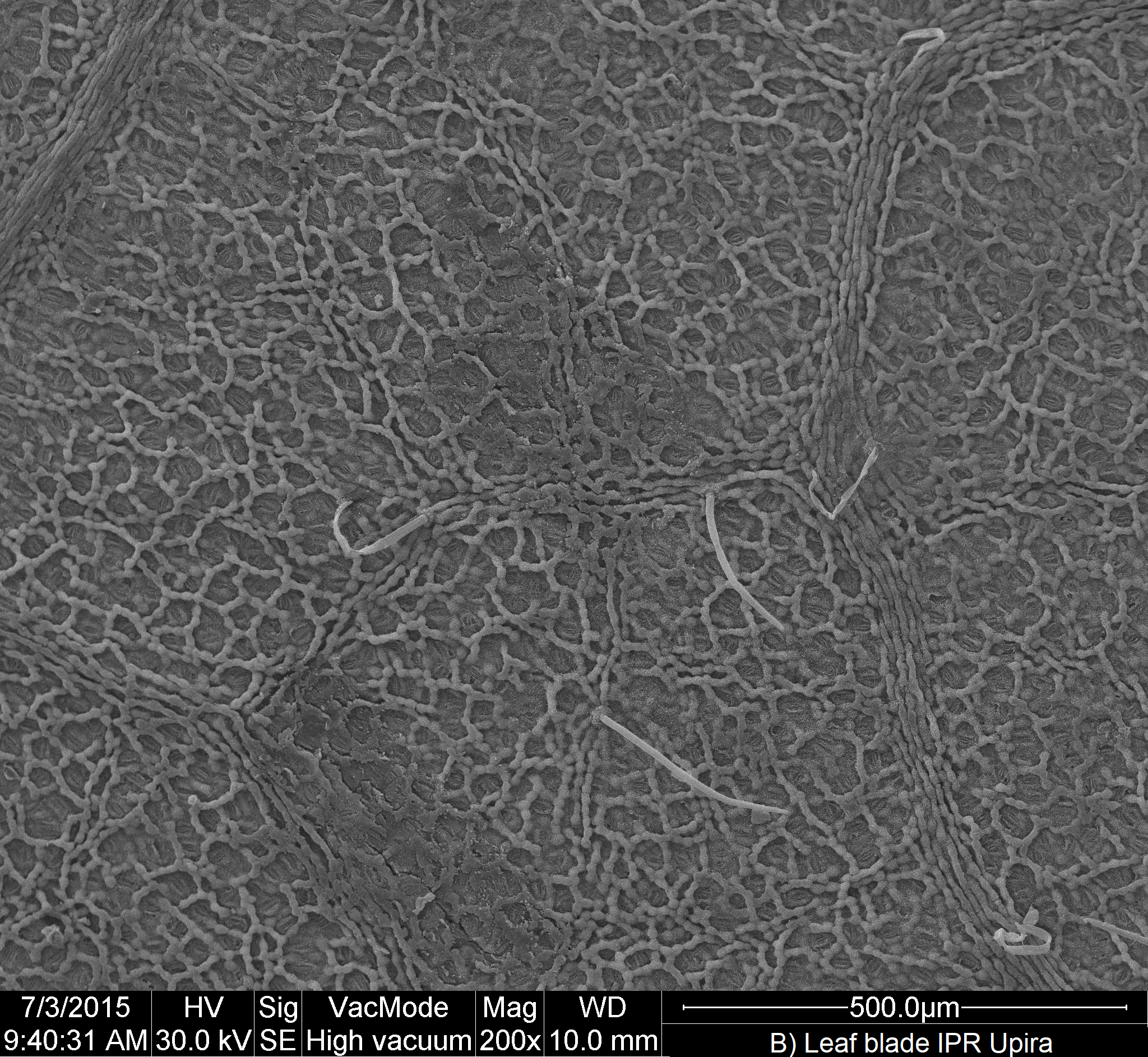

Supplement: Supplementary file 1 [file insects-14-00004-s001.zip › File S2/B) Leaf blade IPR Upira - Superior third portion leaf.tif]

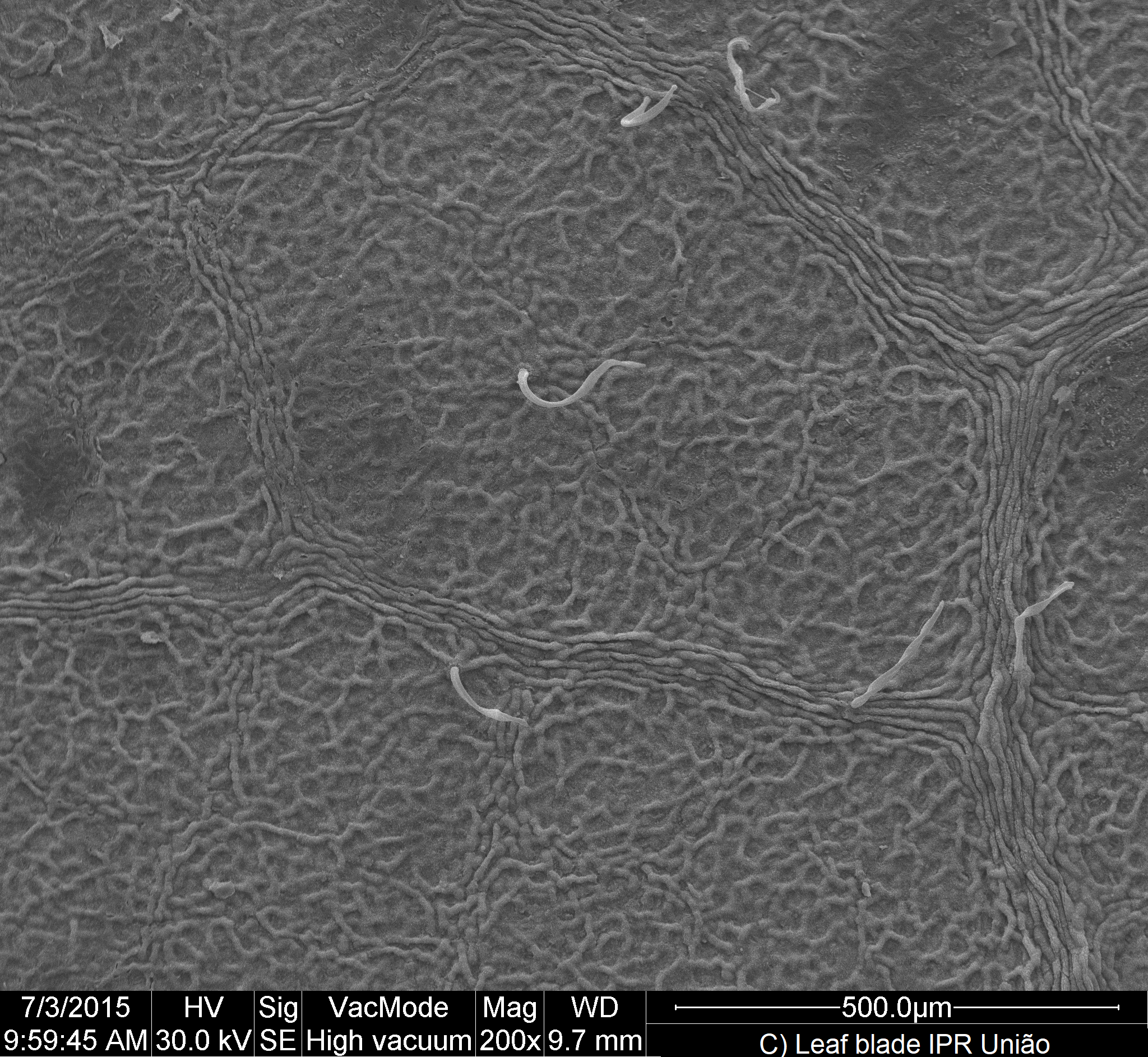

Supplement: Supplementary file 1 [file insects-14-00004-s001.zip › File S2/C) Leaf blade IPR Uni╞o - Superior third portion leaf.tif]

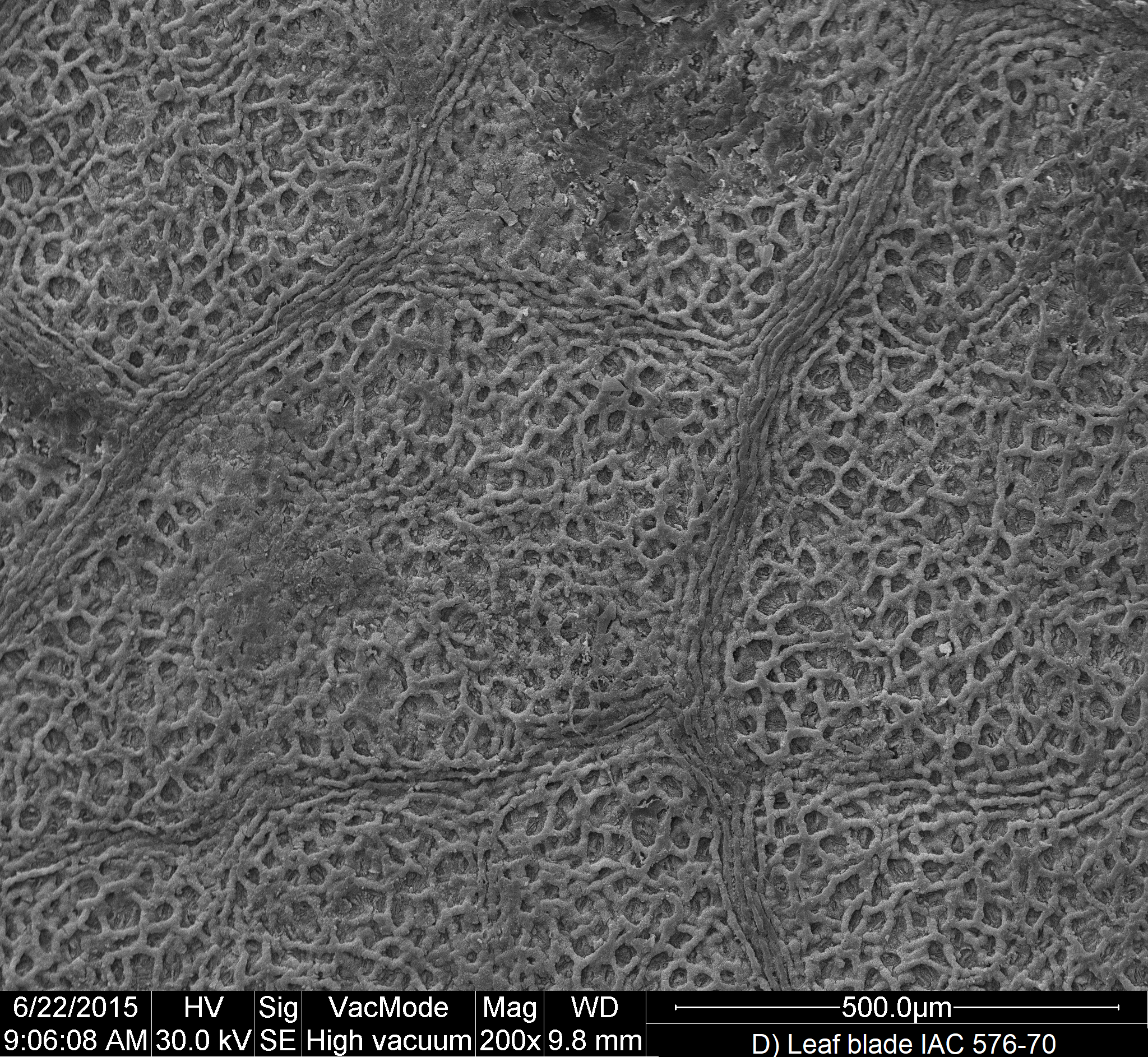

Supplement: Supplementary file 1 [file insects-14-00004-s001.zip › File S2/D) Leaf blade IAC 576-70 - Superior third portion leaf.tif]

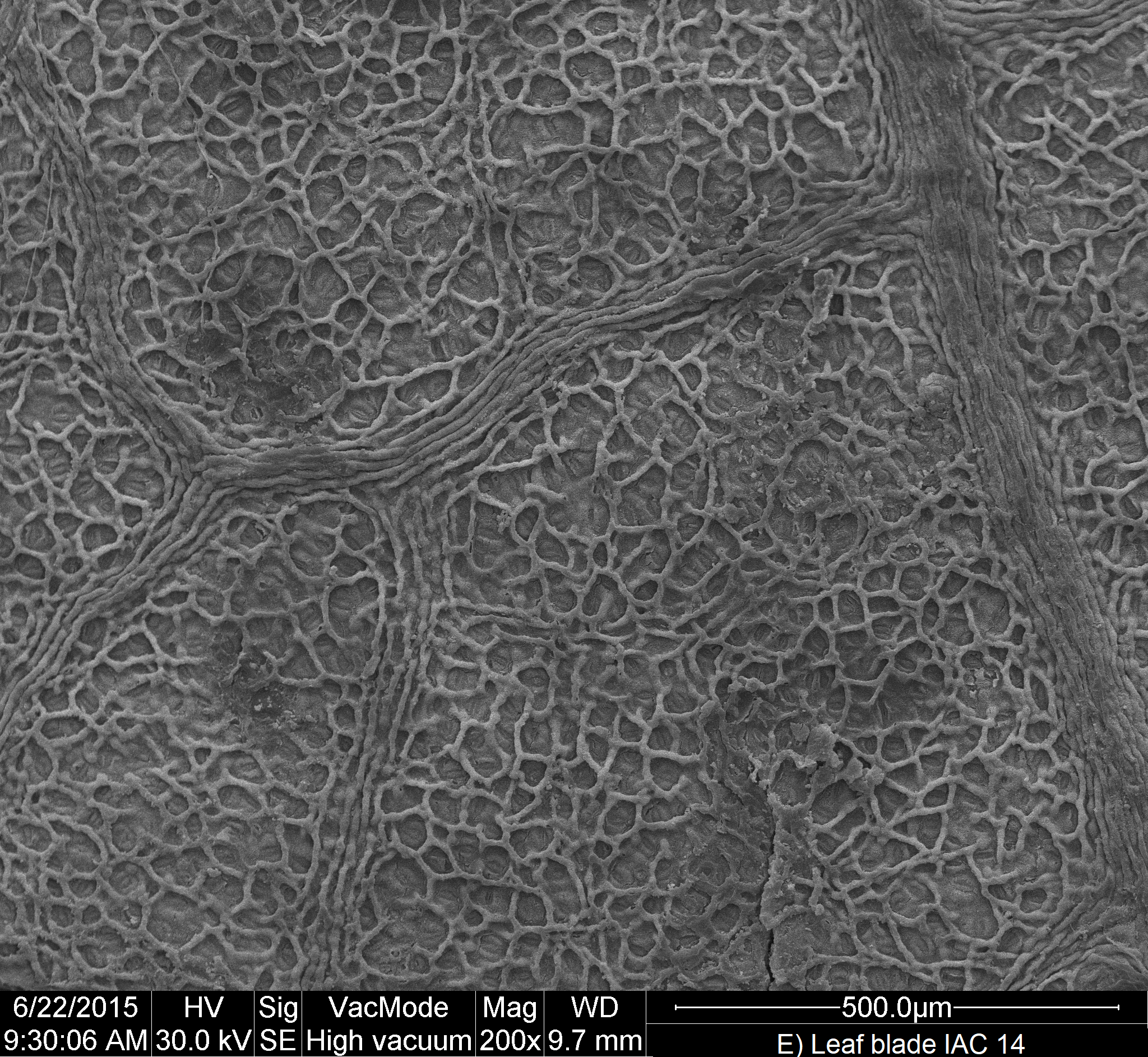

Supplement: Supplementary file 1 [file insects-14-00004-s001.zip › File S2/E) Leaf blade IAC 14 - Superior third portion leaf.tif]

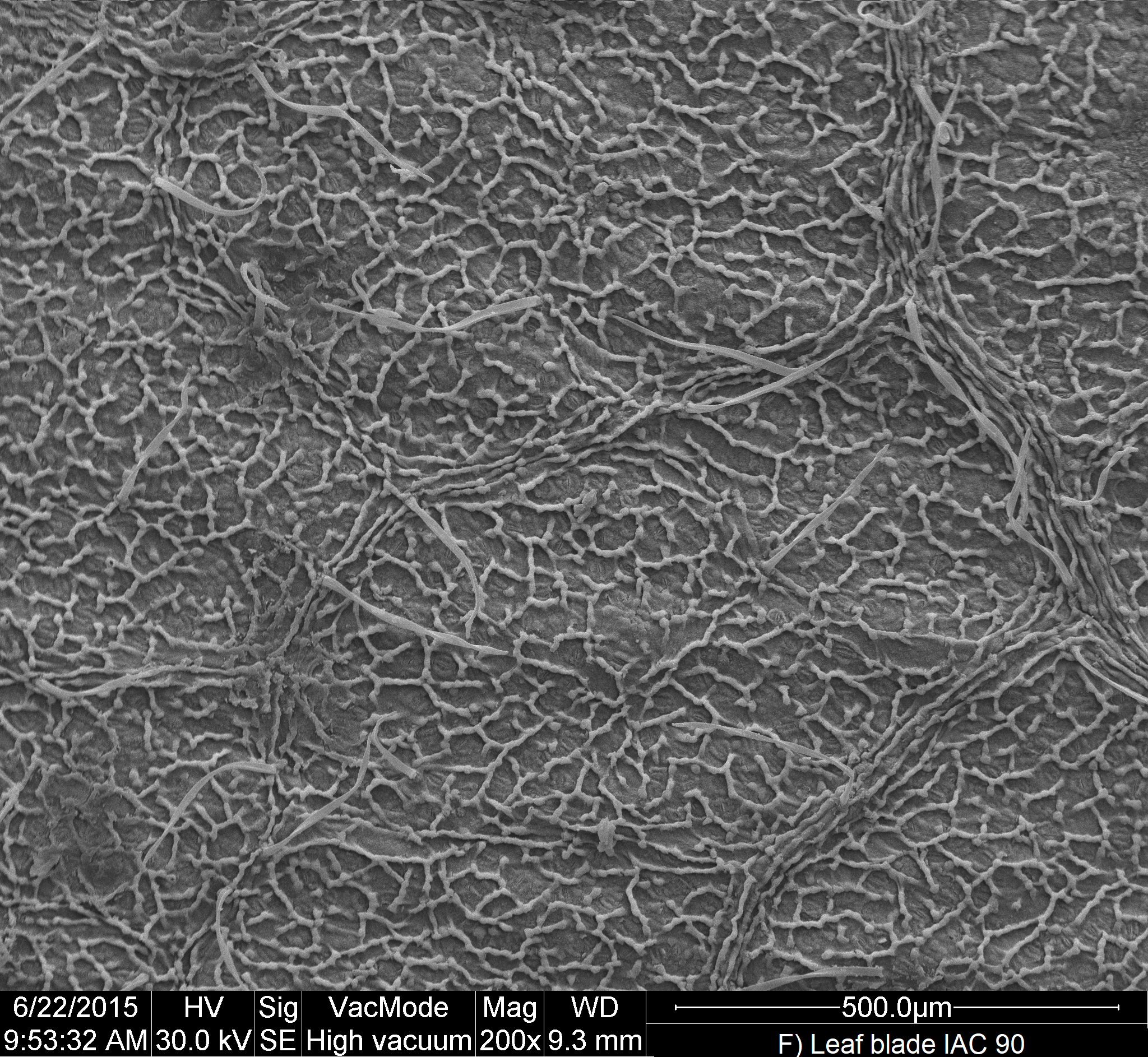

Supplement: Supplementary file 1 [file insects-14-00004-s001.zip › File S2/F) Leaf blade IAC 90 - Superior third portion leaf.tif]

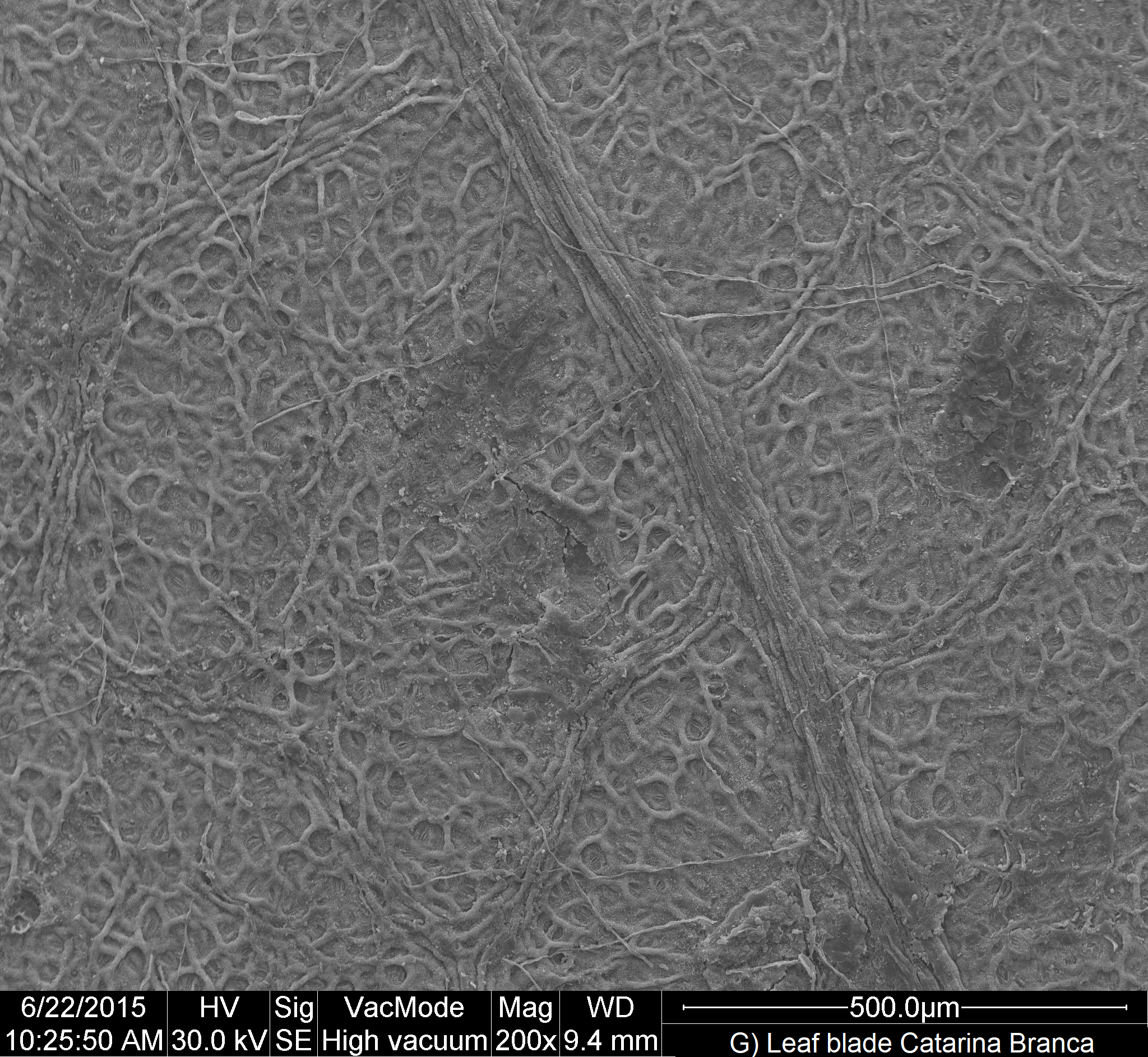

Supplement: Supplementary file 1 [file insects-14-00004-s001.zip › File S2/G) Leaf blade Catarina Branca - Superior third portion leaf.tif]

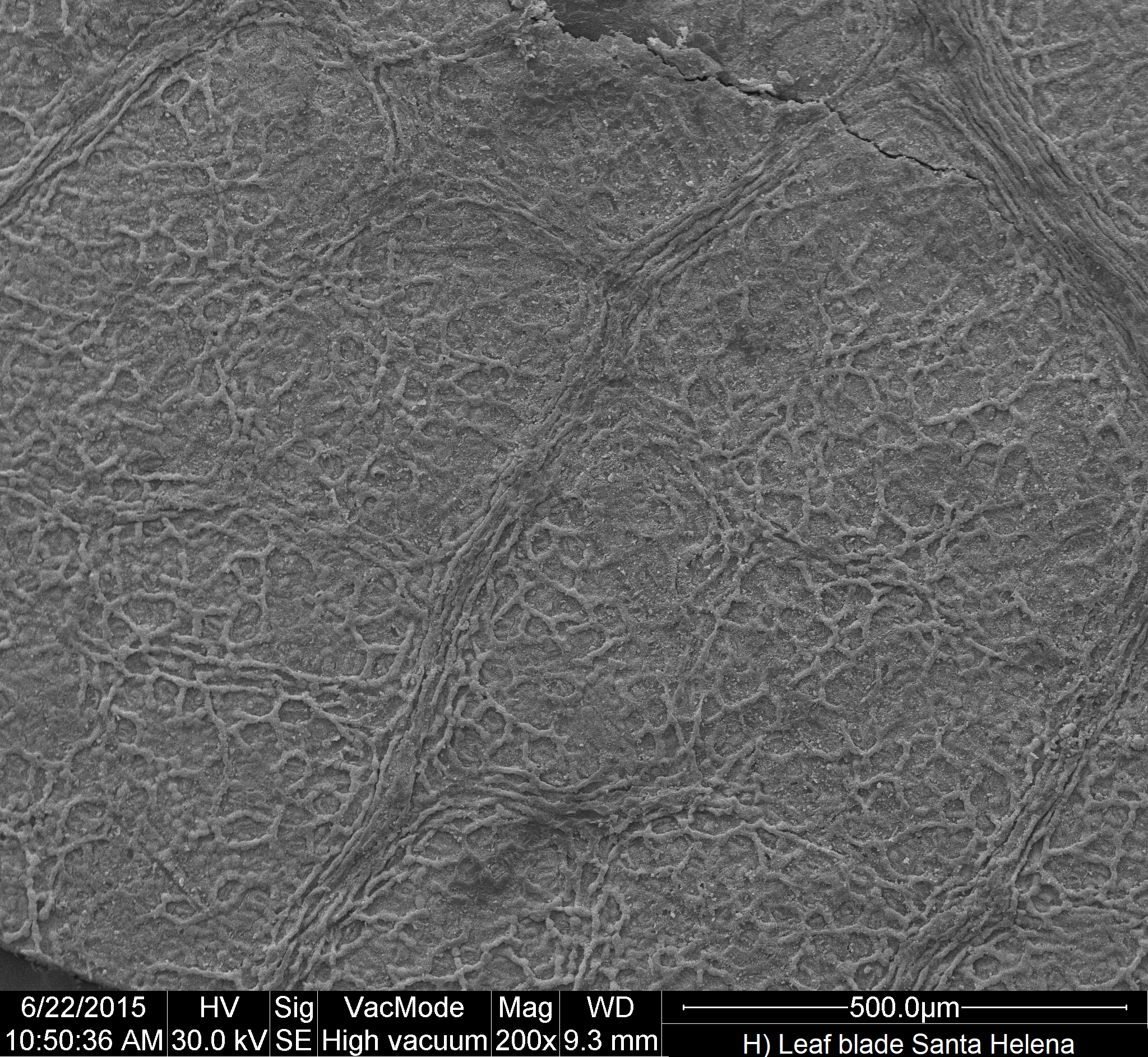

Supplement: Supplementary file 1 [file insects-14-00004-s001.zip › File S2/H) Leaf blade Santa Helena - Superior third portion leaf.tif]

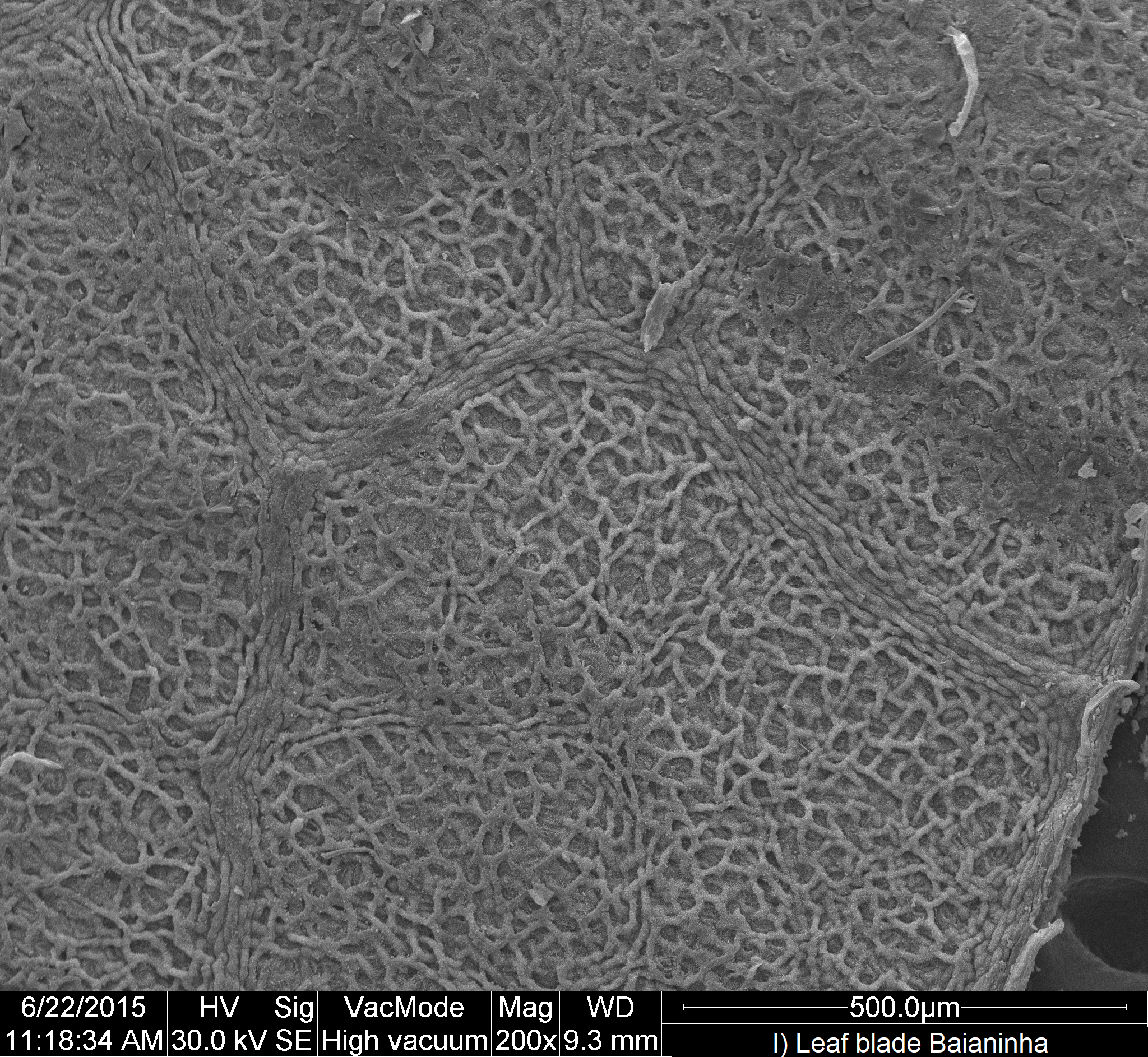

Supplement: Supplementary file 1 [file insects-14-00004-s001.zip › File S2/I) Leaf blade Baianinha - Superior third portion leaf.tif]

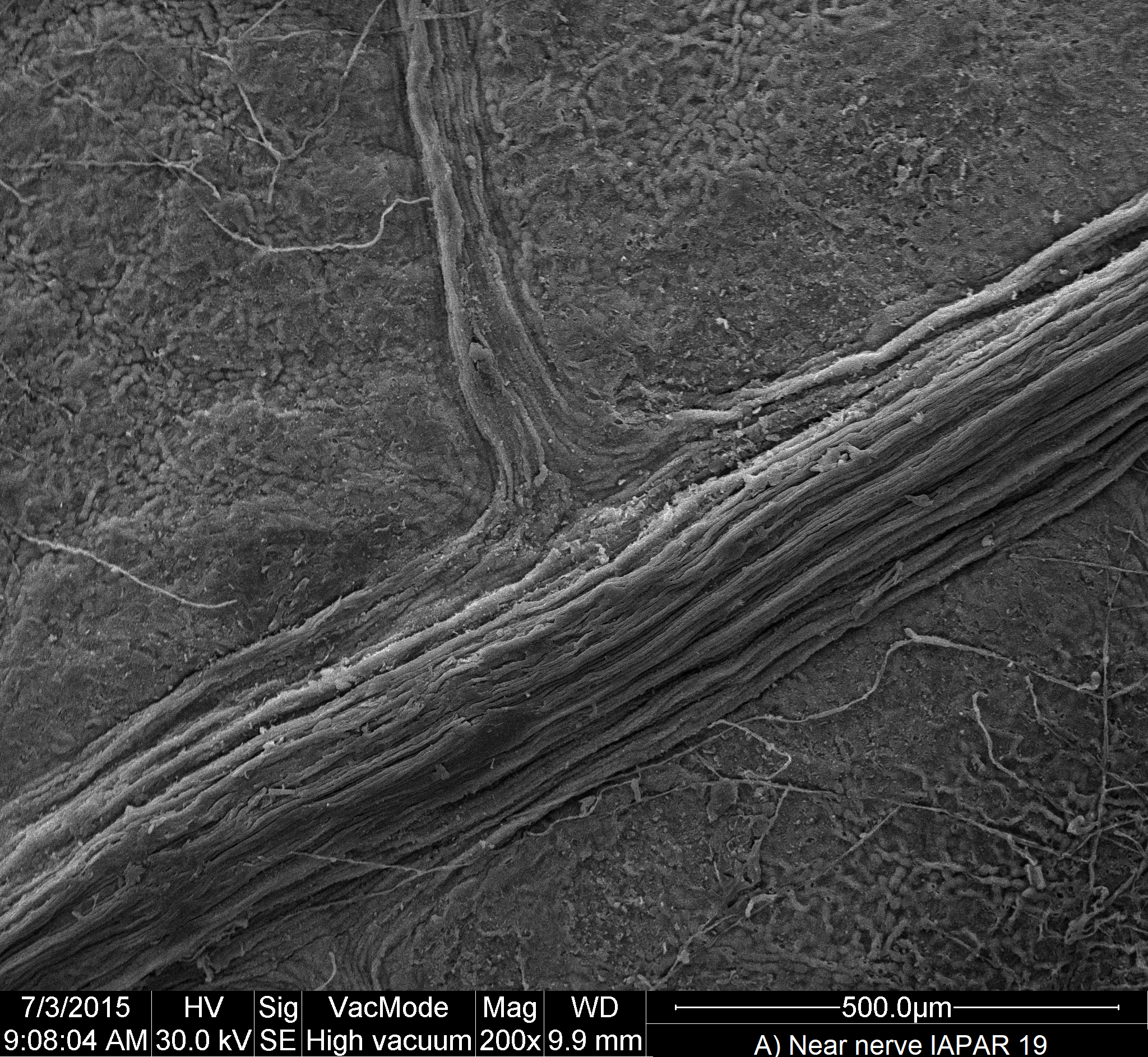

Supplement: Supplementary file 1 [file insects-14-00004-s001.zip › File S3/A) Near nerve IAPAR 19 - Shoot leaf.tif]

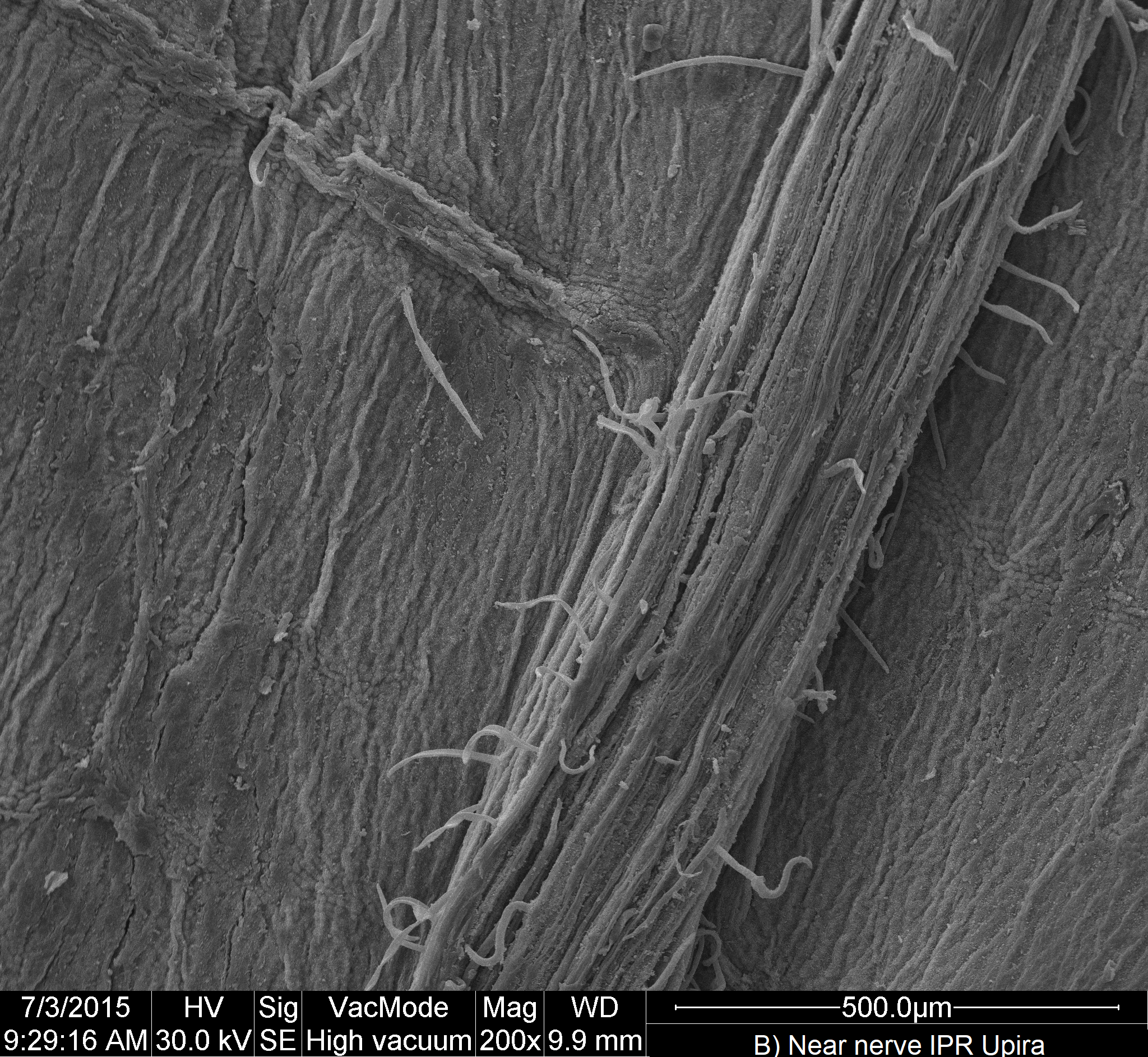

Supplement: Supplementary file 1 [file insects-14-00004-s001.zip › File S3/B) Near nerve IPR Upira - Shoot leaf.tif]

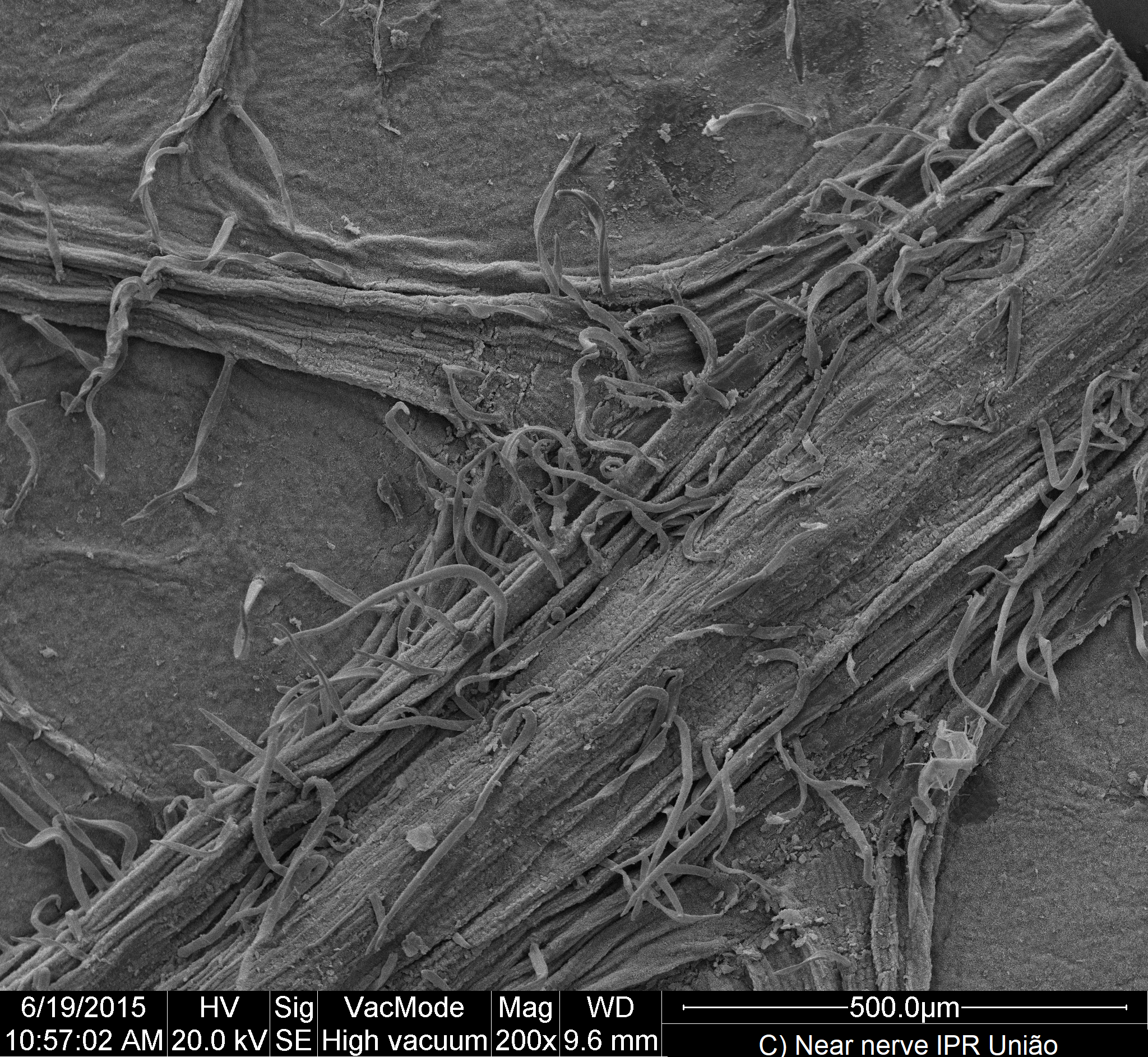

Supplement: Supplementary file 1 [file insects-14-00004-s001.zip › File S3/C) Near nerve IPR Uni╞o - Shoot leaf.tif]

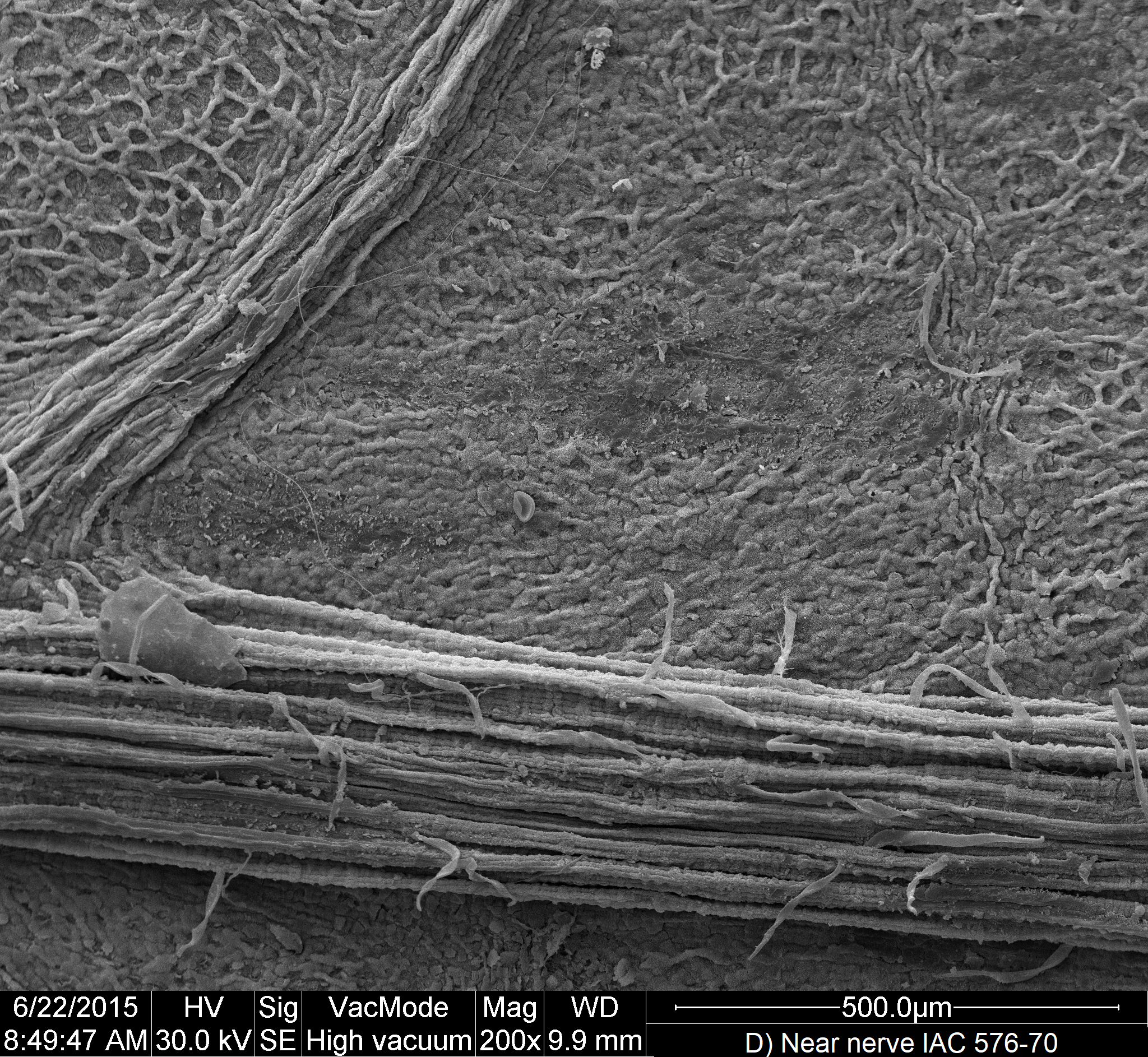

Supplement: Supplementary file 1 [file insects-14-00004-s001.zip › File S3/D) Near nerve IAC 576-70 - Shoot leaf.tif]

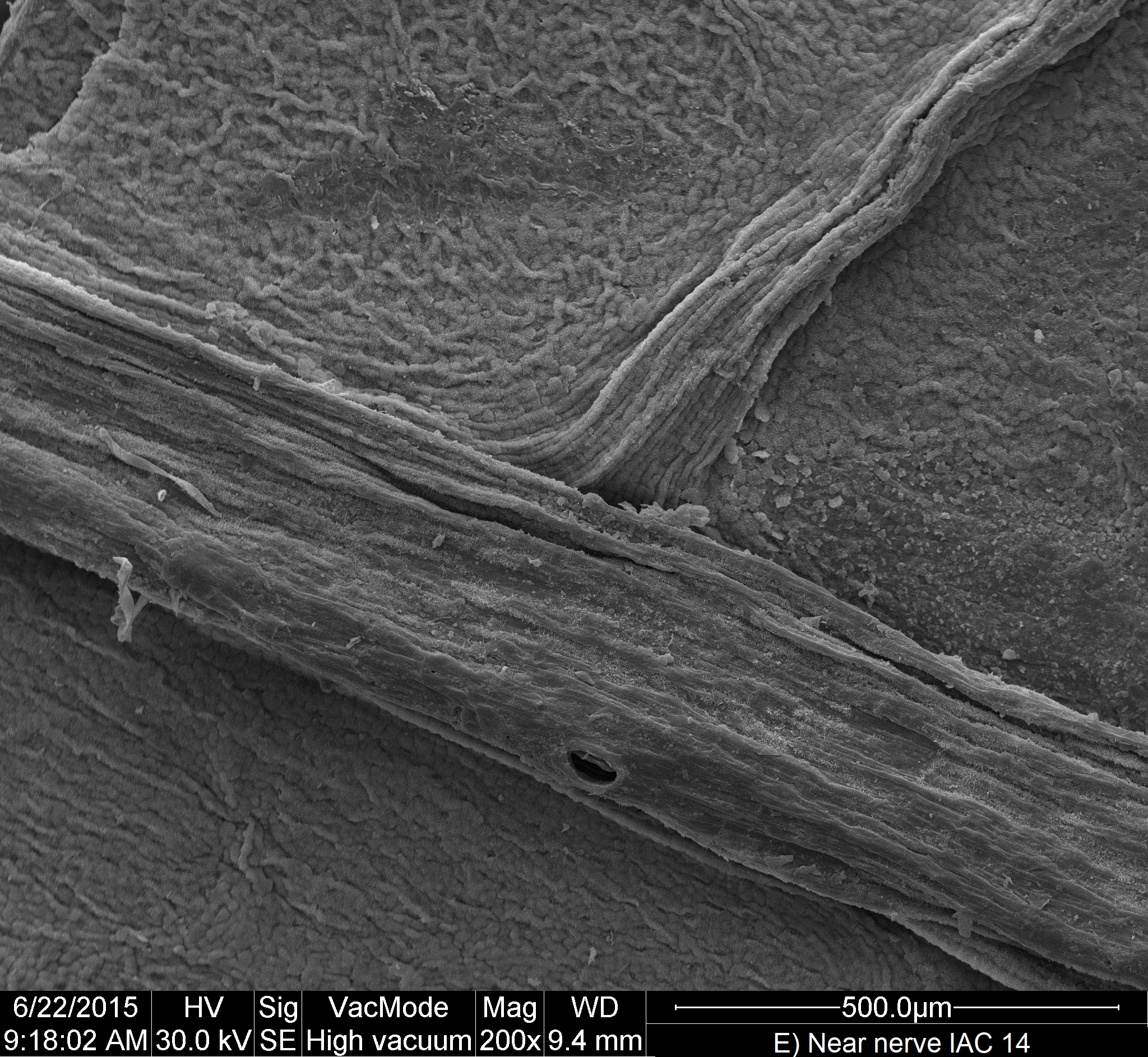

Supplement: Supplementary file 1 [file insects-14-00004-s001.zip › File S3/E) Near nerve IAC 14 - Shoot leaf.tif]

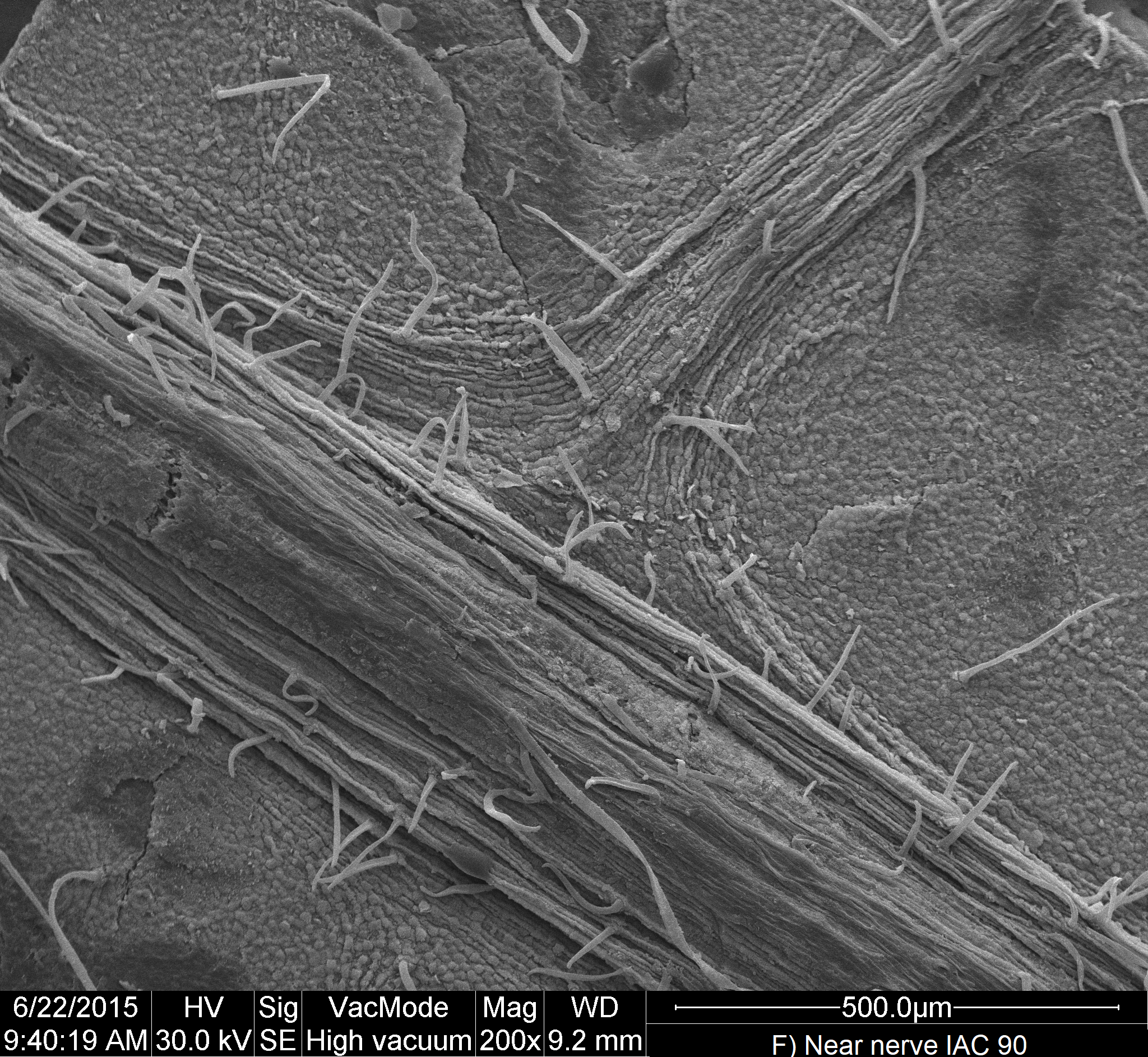

Supplement: Supplementary file 1 [file insects-14-00004-s001.zip › File S3/F) Near nerve IAC 90 - Shoot leaf.tif]

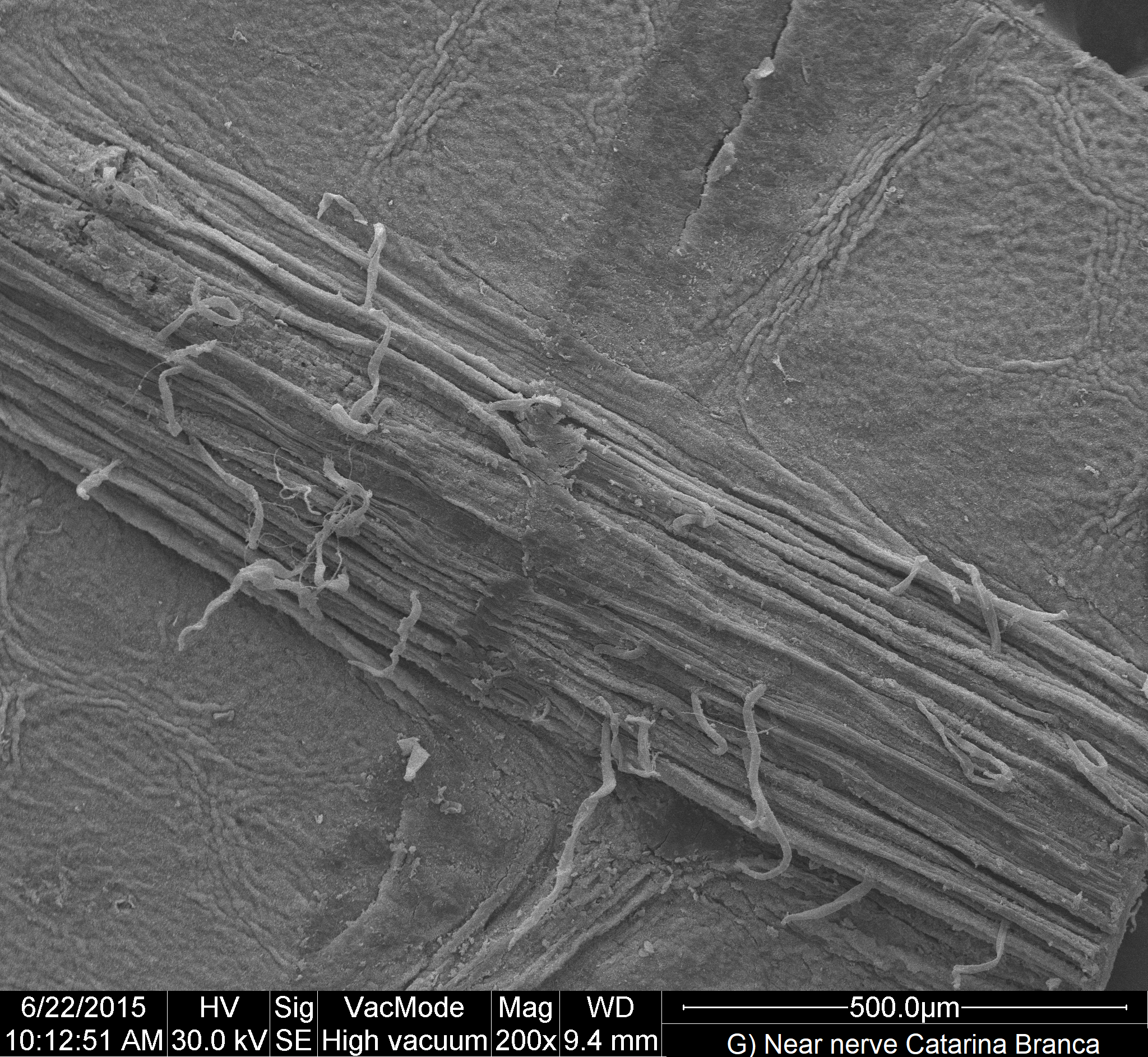

Supplement: Supplementary file 1 [file insects-14-00004-s001.zip › File S3/G) Near nerve Catarina Branca - Shoot leaf.tif]

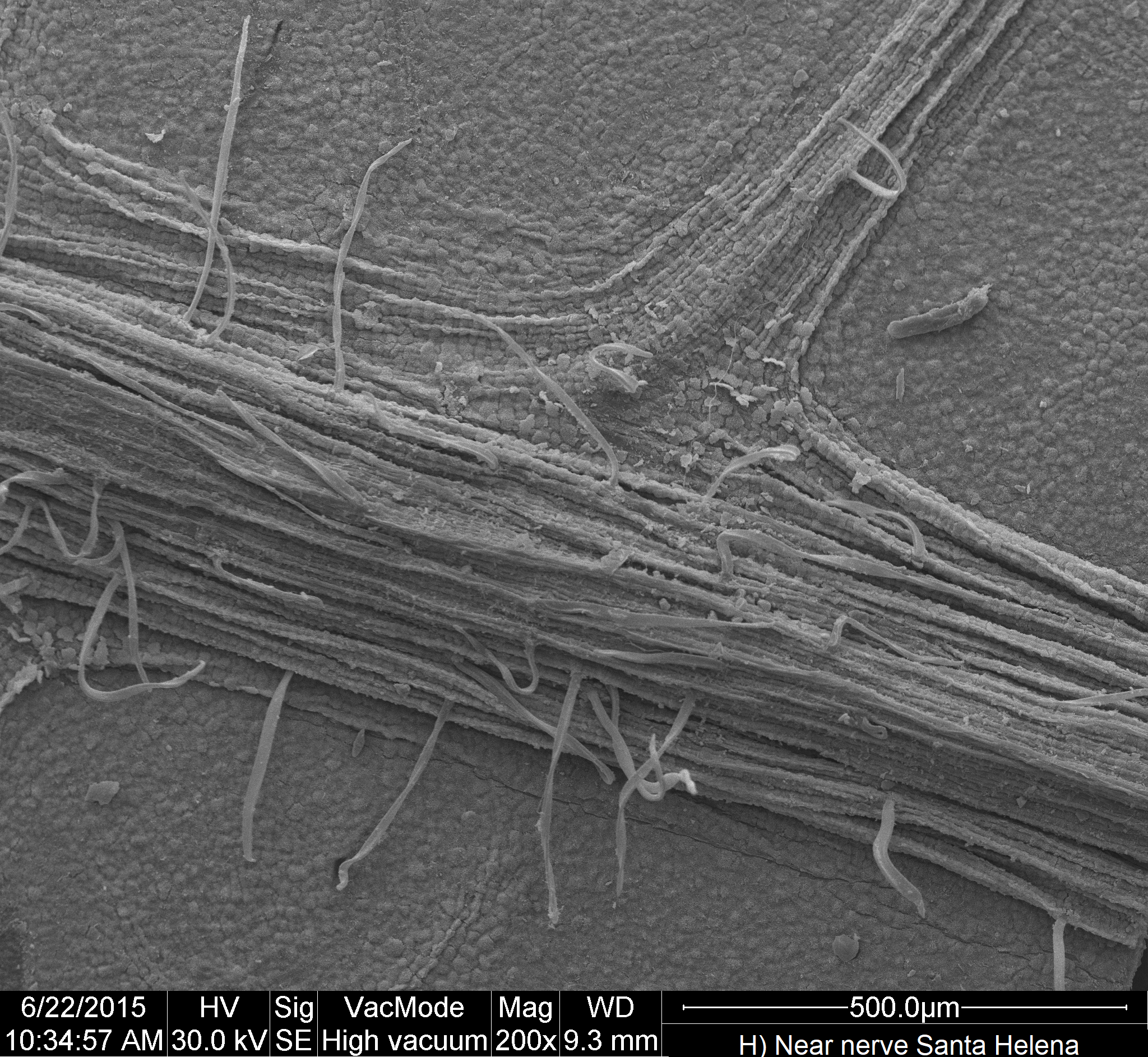

Supplement: Supplementary file 1 [file insects-14-00004-s001.zip › File S3/H) Near nerve Santa Helena - Shoot leaf.tif]

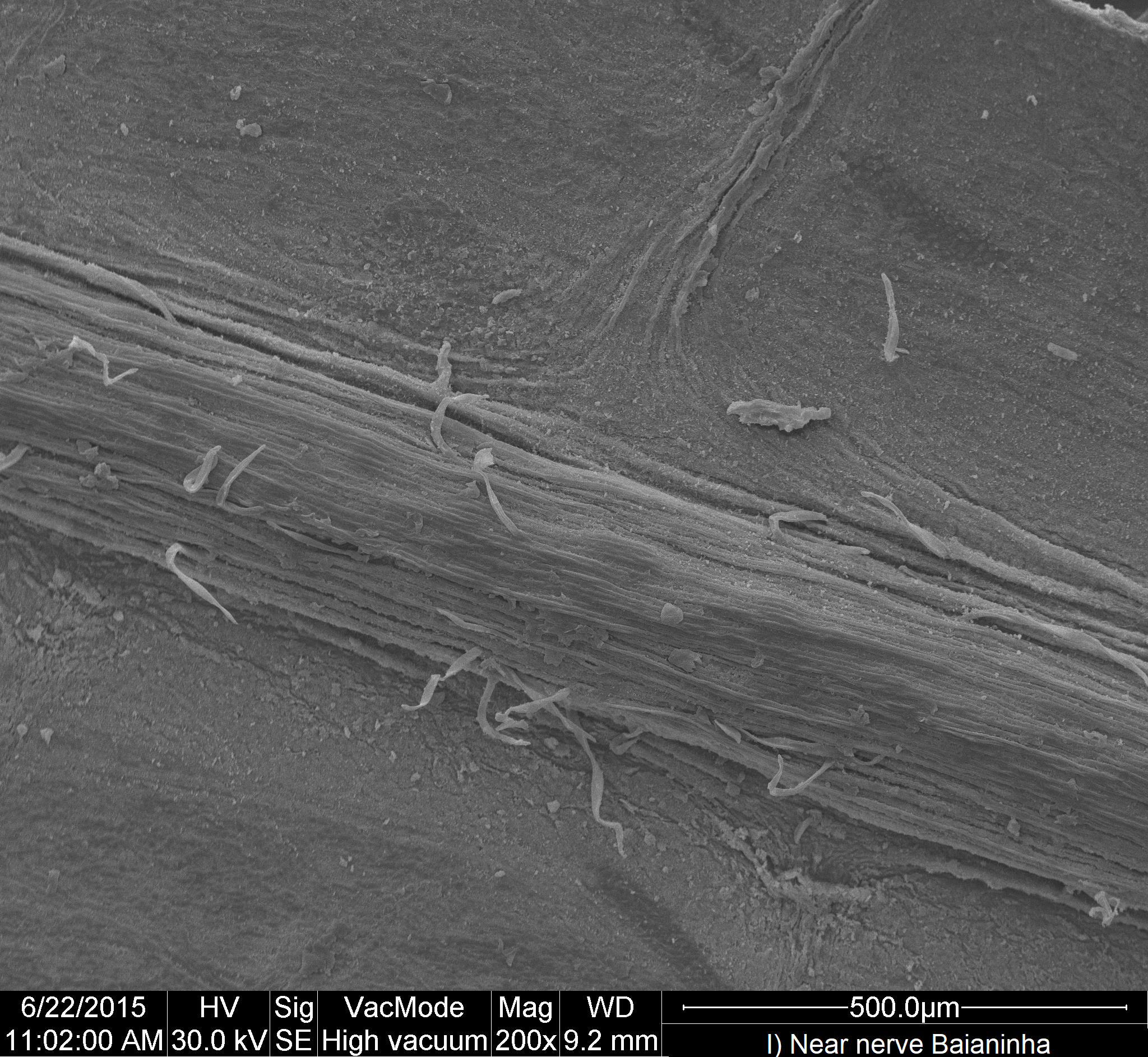

Supplement: Supplementary file 1 [file insects-14-00004-s001.zip › File S3/I) Near nerve Baianinha - Shoot leaf.tif]

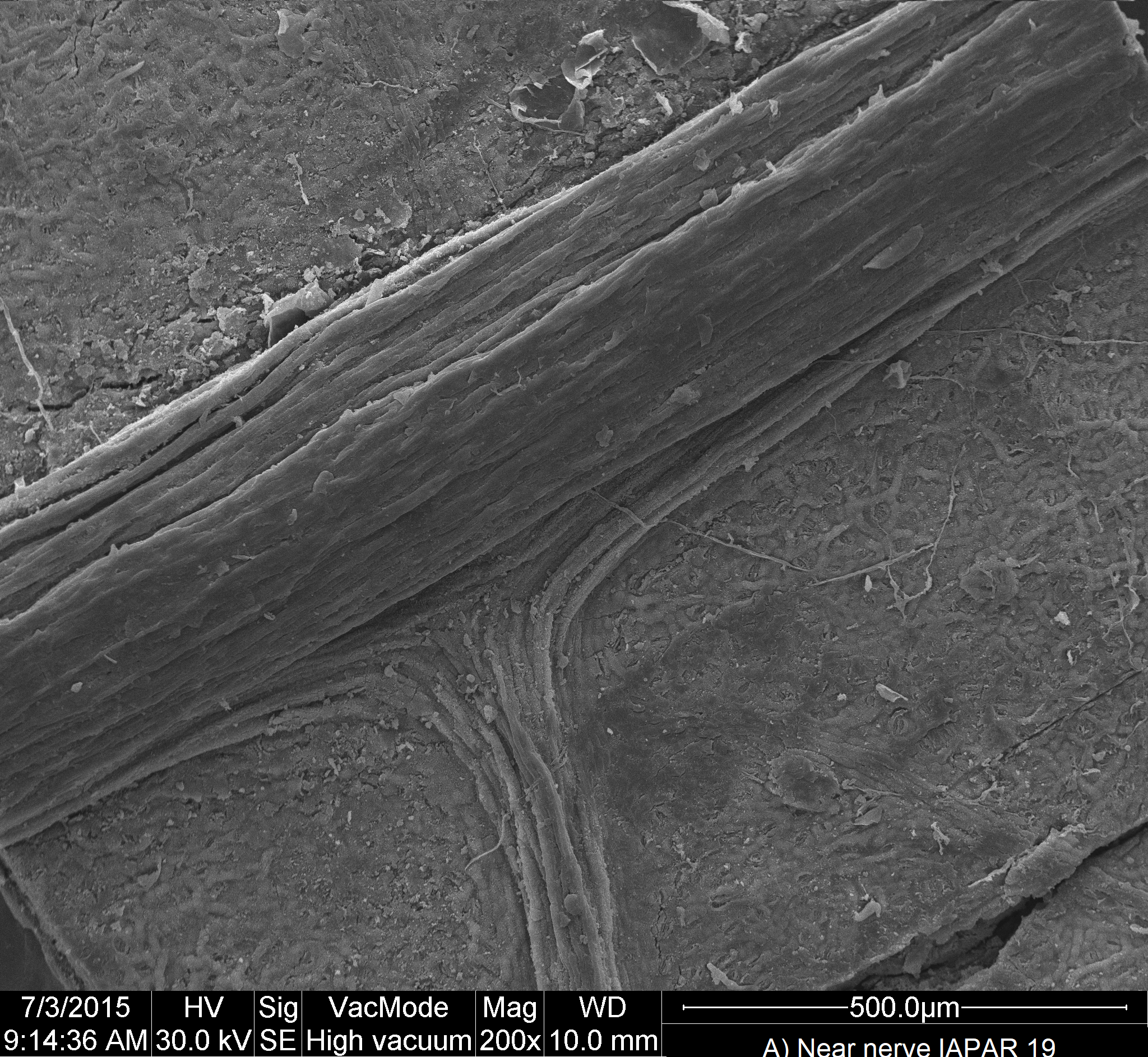

Supplement: Supplementary file 1 [file insects-14-00004-s001.zip › File S4/A) Near nerve IAPAR 19 - Superior third portion leaf.tif]

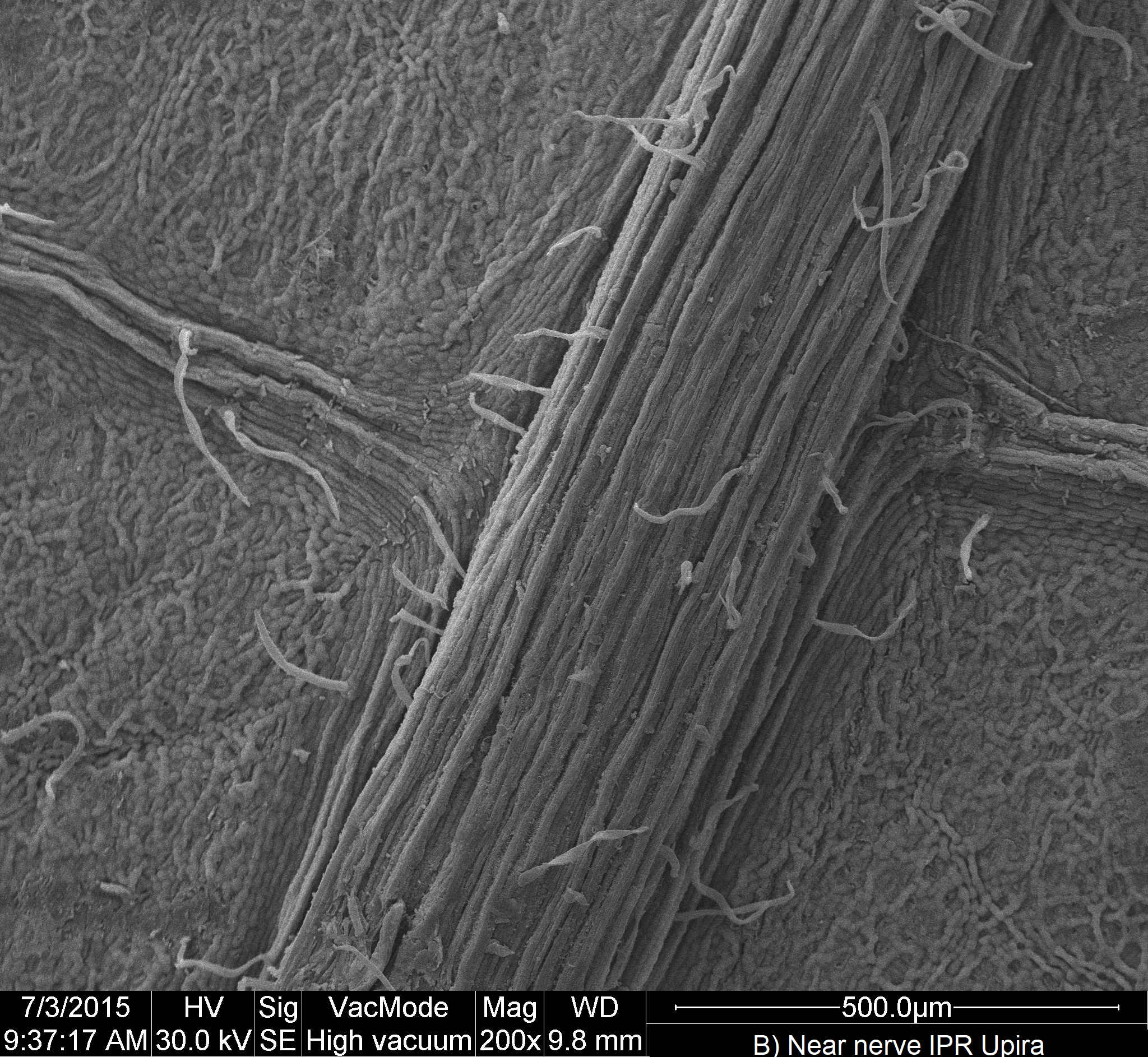

Supplement: Supplementary file 1 [file insects-14-00004-s001.zip › File S4/B) Near nerve IPR Upira - Superior third portion leaf.tif]

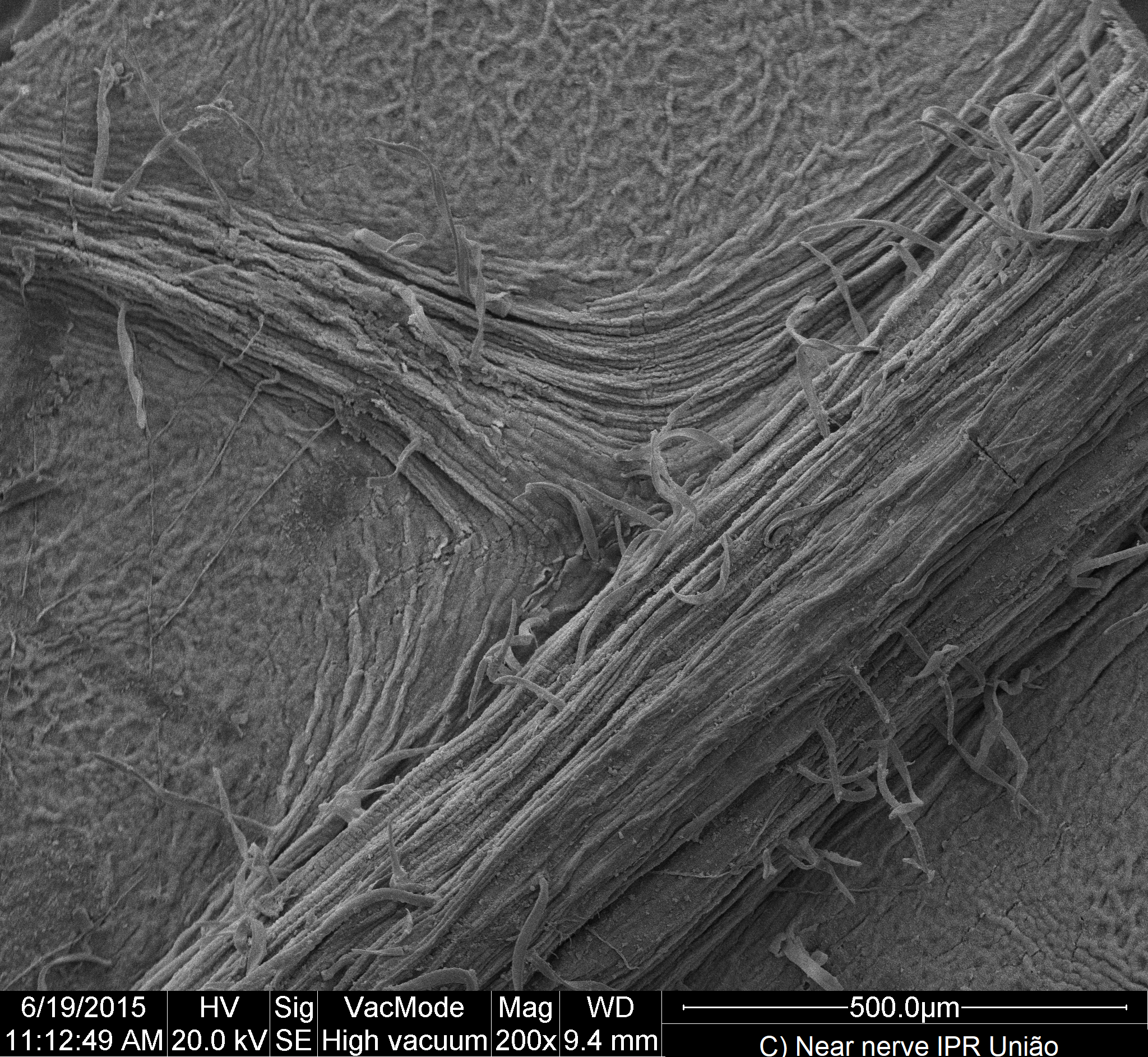

Supplement: Supplementary file 1 [file insects-14-00004-s001.zip › File S4/C) Near nerve IPR Uni╞o - Superior third portion leaf.tif]

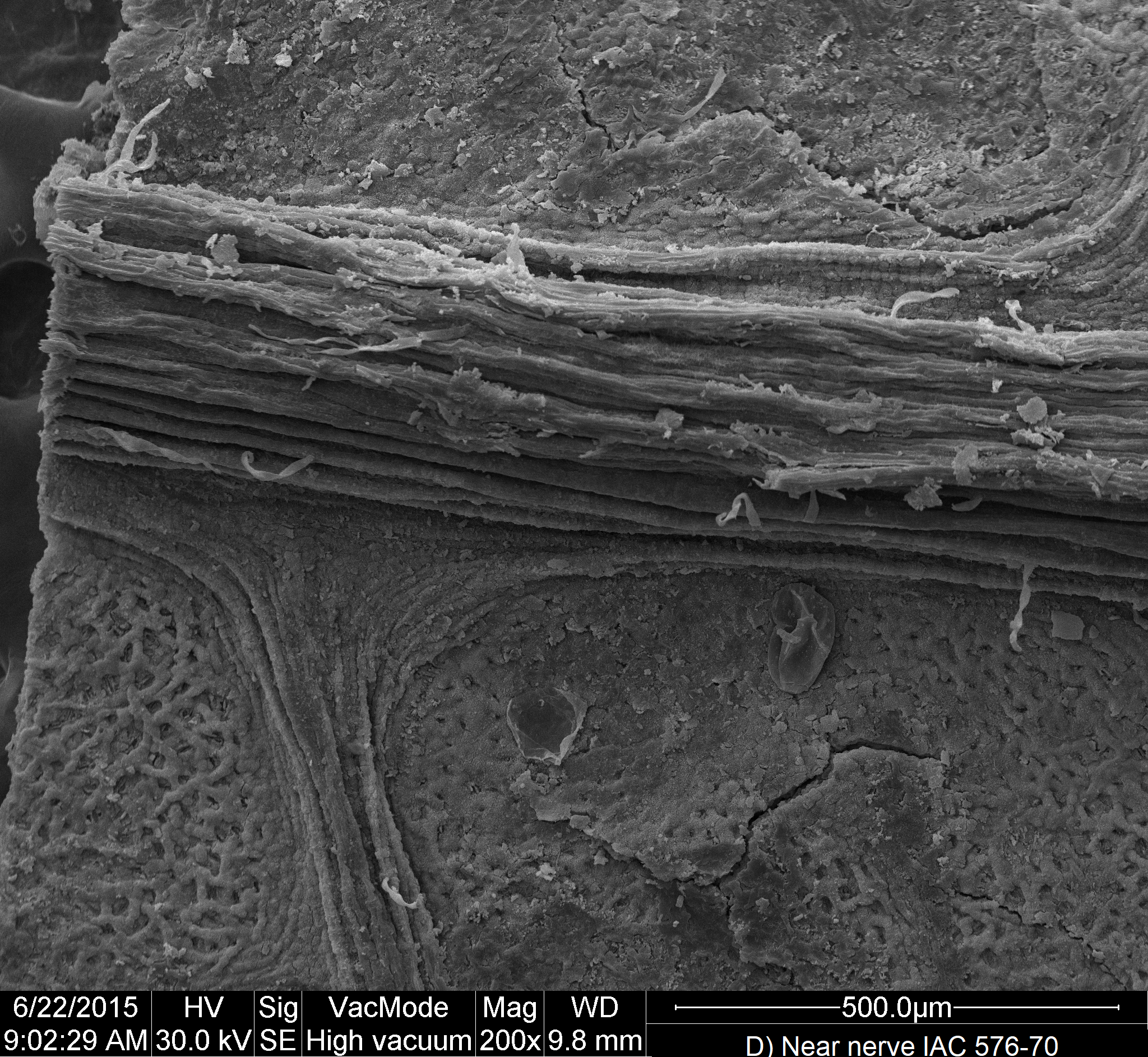

Supplement: Supplementary file 1 [file insects-14-00004-s001.zip › File S4/D) Near nerve IAC 576-70 - Superior third portion leaf.tif]

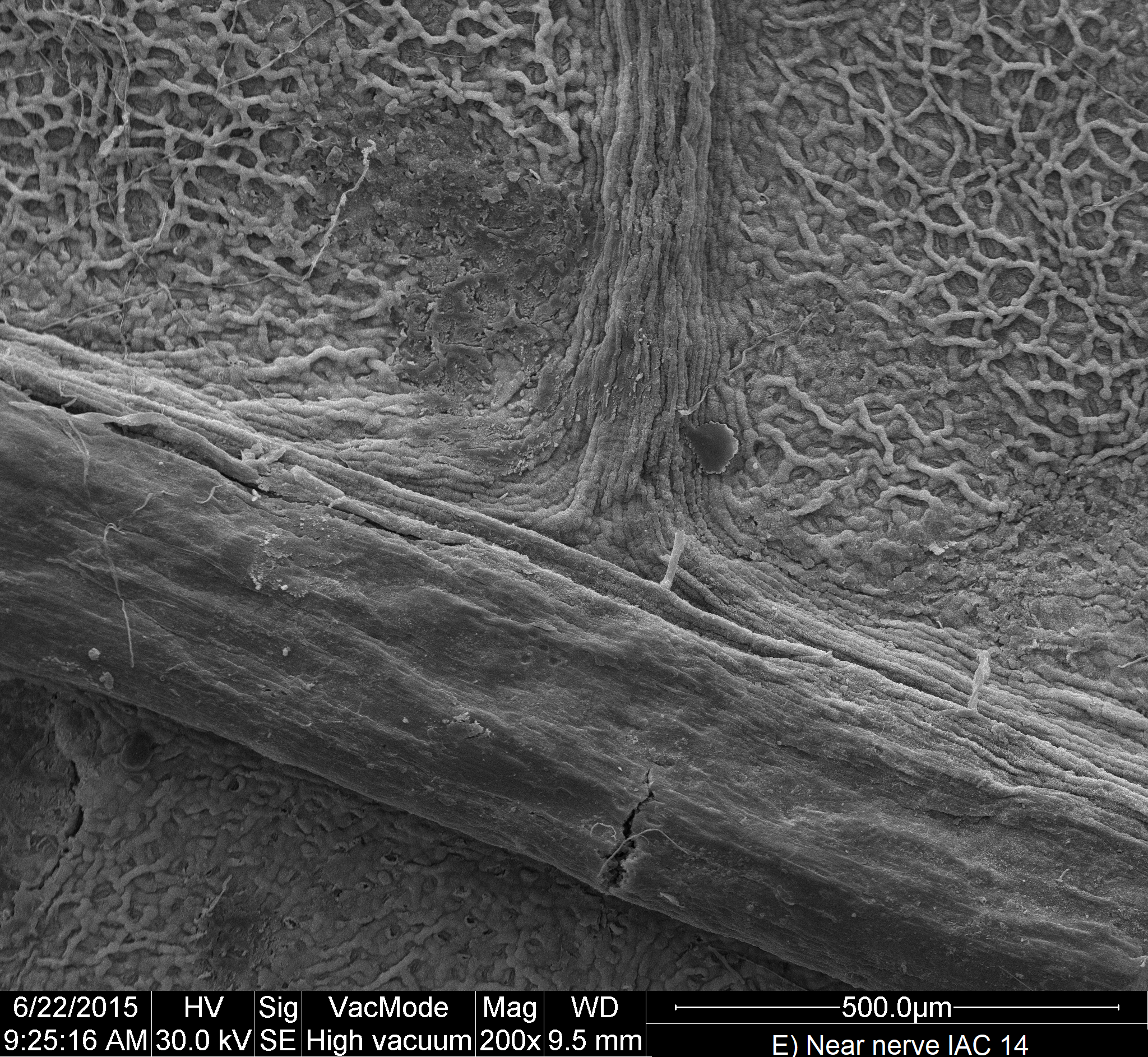

Supplement: Supplementary file 1 [file insects-14-00004-s001.zip › File S4/E) Near nerve IAC 14 - Superior third portion leaf.tif]

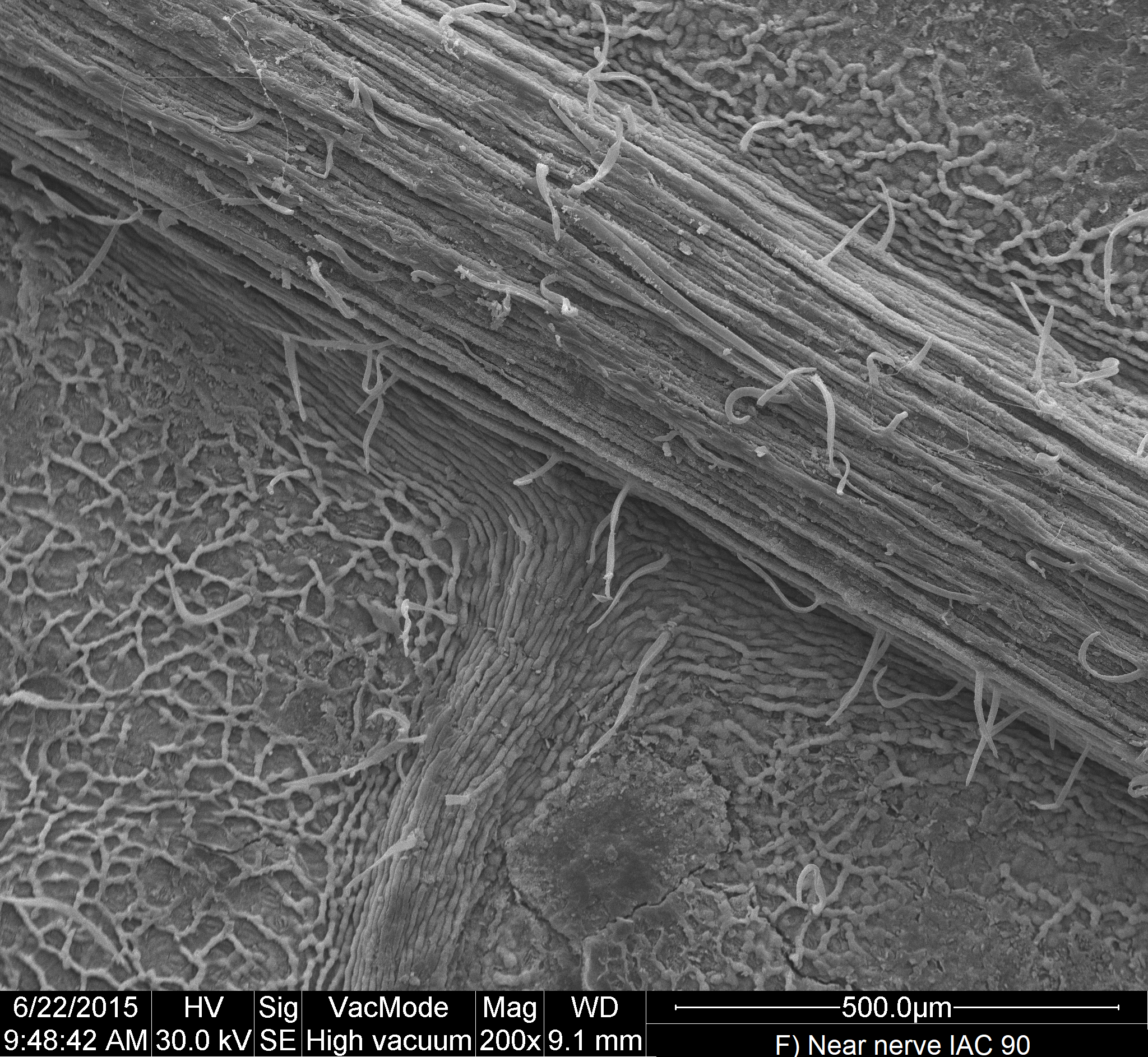

Supplement: Supplementary file 1 [file insects-14-00004-s001.zip › File S4/F) Near nerve IAC 90 - Superior third portion leaf.tif]

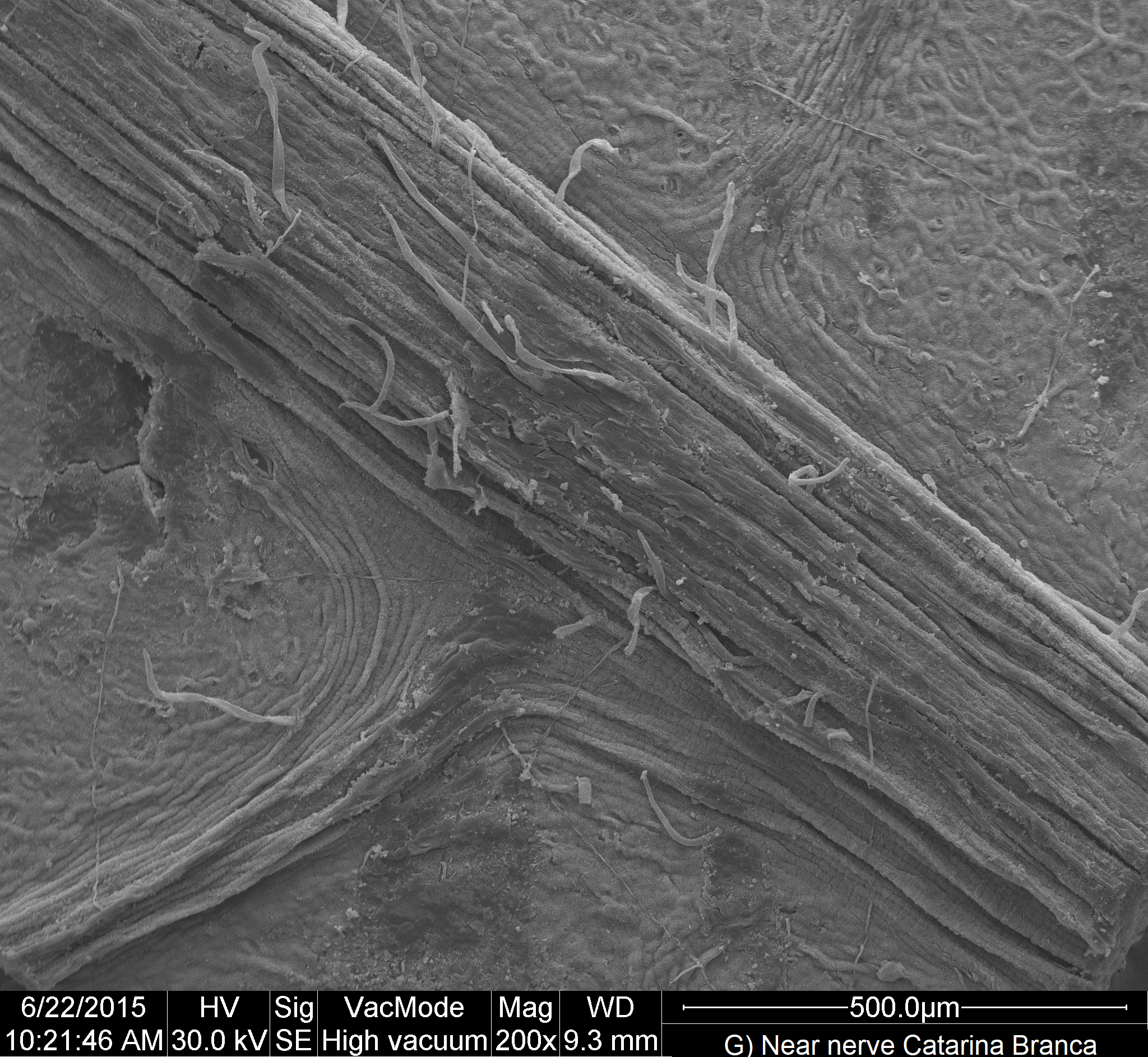

Supplement: Supplementary file 1 [file insects-14-00004-s001.zip › File S4/G) Near nerve Catarina Branca - Superior third portion leaf.tif]

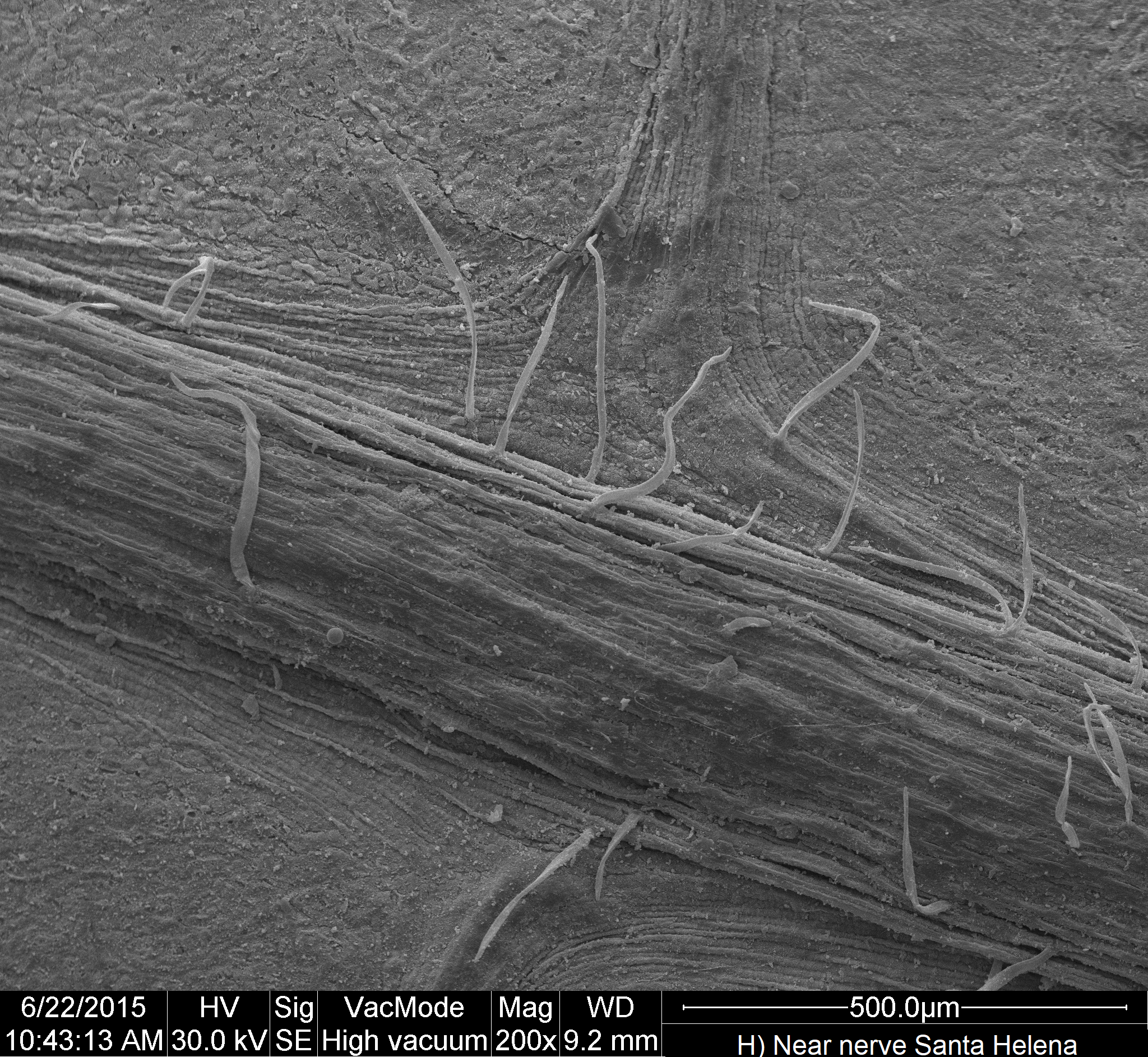

Supplement: Supplementary file 1 [file insects-14-00004-s001.zip › File S4/H) Near nerve Santa Helena - Superior third portion leaf.tif]

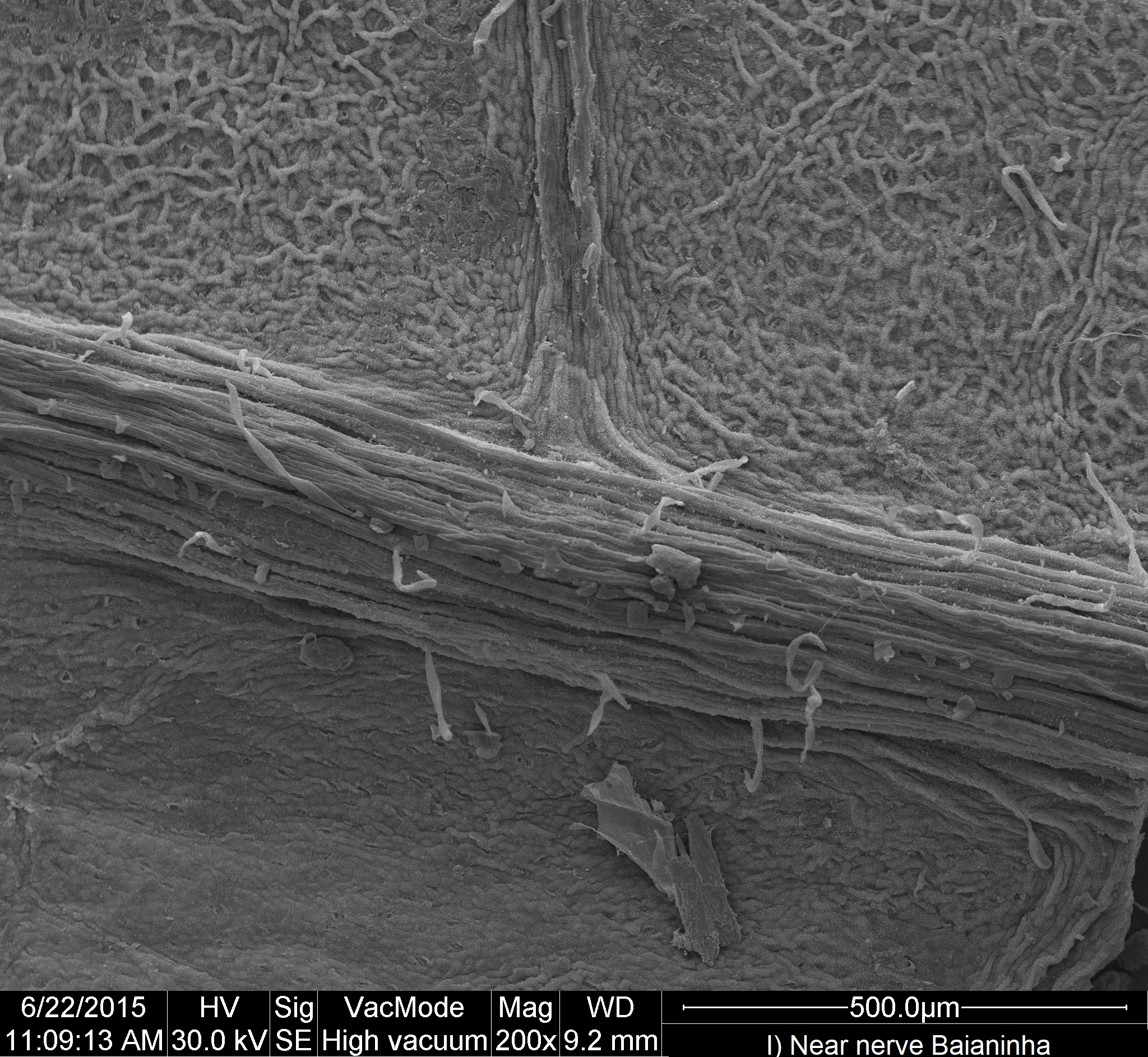

Supplement: Supplementary file 1 [file insects-14-00004-s001.zip › File S4/I) Near nerve Baianinha - Superior third portion leaf.tif]
